# Mobilome-driven partitions of the resistome in *Salmonella*

**DOI:** 10.1128/msystems.00883-23

**Published:** 2023-10-19

**Authors:** Chenghao Jia, Zining Wang, Chenghu Huang, Lin Teng, Haiyang Zhou, Hongli An, Sihao Liao, Yuhao Liu, Linlin Huang, Biao Tang, Min Yue

**Affiliations:** 1 Department of Veterinary Medicine, Institute of Preventive Veterinary Sciences, Zhejiang University College of Animal Sciences, Hangzhou, China; 2 Zhejiang Provincial Key Laboratory of Preventive Veterinary Medicine, Hangzhou, China; 3 Hainan Institute of Zhejiang University, Sanya, China; 4 State Key Laboratory for Managing Biotic and Chemical Threats to the Quality and Safety of Agro-Products, Institute of Agro-Product Safety and Nutrition, Zhejiang Academy of Agricultural Sciences, Hangzhou, China; 5 State Key Laboratory for Diagnosis and Treatment of Infectious Diseases, National Clinical Research Center for Infectious Diseases, National Medical Center for Infectious Diseases, The First Affiliated Hospital, College of Medicine, Zhejiang University, Hangzhou, China; Wageningen University, Wageningen, the Netherlands

**Keywords:** mobile genetic elements, resistome, complete genome sequence, *Salmonella*, horizontal gene transfer

## Abstract

Mobile genetic elements (MGEs) or mobilomes promote the mobilization and dissemination of antimicrobial resistance genes (ARGs), serving as critical drivers for antimicrobial resistance (AMR) accumulation, interaction, and persistence. However, systematic and quantitative evaluations of the role of mobilome in spreading resistome in a bacterial pathogen remain unaddressed, partially due to the lack of closed genomes. Here, we examined MGEs across 1,817 *Salmonella* isolates with complete genomic sequences from 58 countries between 1911 and 2022. We found the plasmid harboring 69.8% ARGs to be the largest ARG reservoir, correlated with serovar-based evolution in most *Salmonella* lineages. Prophages, specifically RCS47 and SJ46, play a crucial role in the plasmids’ plasticity and the acquisition of ARGs. Furthermore, distinct ARG accumulation, including resistance toward last-resort antibiotics, exhibited an MGE-favored manner. Certain socioeconomic and ecological factors, as additional layers of mediators, are associated with the preferential distribution of MGE-mediated ARGs in *Salmonella*. Collectively, this study demonstrated an uncharted knowledge of the segmentation of *Salmonella* resistome driven by mobilome, elucidating dynamic drivers and distinct mediators for resistome development that are of immediate relevance for targeted interventions.

**IMPORTANCE:**

Antimicrobial resistance (AMR) has become a significant global challenge, with an estimated 10 million deaths annually by 2050. The emergence of AMR is mainly attributed to mobile genetic elements (MGEs or mobilomes), which accelerate wide dissemination among pathogens. The interaction between mobilomes and AMR genes (or resistomes) in *Salmonella*, a primary cause of diarrheal diseases that results in over 90 million cases annually, remains poorly understood. The available fragmented or incomplete genomes remain a significant limitation in investigating the relationship between AMR and MGEs. Here, we collected the most extensive closed *Salmonella* genomes (*n* = 1,817) from various sources across 58 countries. Notably, our results demonstrate that resistome transmission between *Salmonella* lineages follows a specific pattern of MGEs and is influenced by external drivers, including certain socioeconomic factors. Therefore, targeted interventions are urgently needed to mitigate the catastrophic consequences of *Salmonella* AMR.

## INTRODUCTION

The ongoing antimicrobial resistance (AMR) crisis poses a major public health challenge for the 21st century (1). Such a crisis was estimated to result in 10 million deaths annually by 2050 if no appropriate intervention was conducted (2). The collection of antimicrobial resistance genes (ARGs), also known as the resistome, represents an essential determinant for AMR establishment, further development, and amplification in the microbial community. Mobile genetic elements (MGEs), so-called mobilome, frequently carried ARGs or virulence determinants that contributed to bacterial fitness (3, 4). It is widely acknowledged that MGEs, or mobilomes, are segments of DNA that encode enzymes and proteins that mediate intercellular mobility of ARGs, offering the host bacterial pathogens competitive advantages in the context of AMR selection pressure (5). Hence, to comprehend the dissemination patterns of AMR in a single bacterial population, systematic and quantitative evaluations of different types of MGEs and assessing their relationship with ARGs are key for understanding the current AMR phenomenon, predicting ongoing AMR development, and ultimately guiding novel mitigation strategies in response to this compelling crisis.

MGEs contain several mechanistic distinct element types, including plasmids (stable and self-replicating entities composed of functional genetic modules that collectively facilitate transmission (6), bacteriophages (prophages, forming viral particles in bacterial and transferring via transduction) (7), transposons (DNA sequences that carry transposase and cargo genes) (8), integrons (elements that encode integrase enzymes, as well as acquisition systems), genomic islands (characterized by large DNA element, frequently over 10 kb, and carry fragments of other mobilome) (9), and insertion sequences (ISs; discrete segments of DNA that promote bacterial genetic rearrangements) (10). Depending on whether the chromosome-carried MGEs can self-transfer through conjugation, integrative and conjugative elements (ICEs) and integrative and mobilizable elements (IMEs) have also been defined (4, 11). These elements are arranged at multiple complexes, nested levels, like matryoshka dolls (12).

Advances in whole-genome sequencing (WGS) technology have facilitated the identification of MGEs and related ARGs among bacterial communities (^13–15^^13^–15). However, acquiring contextual information about the co-location of ARGs with MGEs remains a technical challenge (16). Correlation-based analyses and contigs assembled from short reads are commonly employed today. Nevertheless, the findings of correlation-based analyses are frequently uncertain as the identified relationships between ARGs and MGEs may be influenced by unaccounted variables (17, 18). Furthermore, the assembly of short reads presents challenges in accurately determining the exact location of MGEs, mainly due to MGEs’ repetitive nature or when flanked by repetitive ISs (19). The utilization of long-read technology enables the direct identification of the co-location between MGEs and ARGs, serving as an ultimate means to address gaps in our understanding of such relationships.

Despite extensive research on MGEs, the distribution of MGEs generally exhibits distinctive patterns in specific pathogens. *Salmonella*, a leading cause of diarrheal diseases, resulting in >90 million cases annually, is an essential subject for pathogenic studies (20). Especially resistant clones emerged, in particular *Salmonella* serovars, which have become a significant public health concern. However, knowledge regarding an overall profile of distinct types of MGEs and their role in disseminating ARGs among *Salmonella* lineages remains unknown, hurdling rational mitigation approaches. Here, by using 1,817 *Salmonella* representing 58 countries covering 1911–2022 with complete genomic sequences, we systematically assessed and quantitatively compared (i) the prevalence of diverse predominant MGEs that mediated ARGs dissemination among *Salmonella* lineages, (ii) the interaction between MGEs and plasmids’ plasticity, (iii) which MGEs mediated the spread of the *bla*_NDM_, *mcr*, and *tet*(X) family ARGs, and (iv) how socioeconomic and ecological factors drive the spread of MGEs harboring ARGs. Altogether, our newly provided knowledge between MGEs and ARGs underscored a targeted intervention strategy to mitigate the AMR crisis in *Salmonella*.

## RESULTS

### Prevalence of MGEs in *Salmonella*

A total of 1,817 *Salmonella* can be divided into seven subspecies and 235 serovars, with different types of MGEs distributed disproportionately (Fig. S1). For plasmids, a total of 1,999 closed sequences were identified. Of them, 1,208 were detected with a known replicon and were further categorized into 82 distinct plasmid types, while the remaining plasmid types were stated as “unknown” (Fig. S2a). The most prevalent plasmid types found were “*IncFIB, IncFII*” (*n* = 204), “*IncI1*” (*n* = 153), “*IncHI2, IncHI2A*” (*n* = 149), “*IncA/C2*” (*n* = 114), and “*IncX1*” (*n* = 93) (Fig. S2b). Furthermore, we constructed a phylogenetic tree to investigate the distribution of “*IncFII/IncFIB*”-type plasmids among *Salmonella*. The phylogenetic tree shows that “*IncFII/IncFIB*”-type plasmids were mainly present in the two dominant serovars: Typhimurium and Enteritidis. However, they were also present in other serovars such as 4,[5],12: i:-, Choleraesuis, etc., indicating that plasmids of the same type can be transferred between different *Salmonella* serovars (Fig. S2c).

*In silico* analyses revealed a total of 17,675 prophage sequences in *Salmonella* genomes, of which 5,651 were intact and 12,024 were defective (Fig. S3a). An explanation for this significantly higher prevalence of defective prophages is that the host is more susceptible to mutations in the intact prophage sequences, which make them inactivated (21). All strains carried defective prophages, with up to 96.4% of *Salmonella* containing more than two defective prophages. On the other hand, intact prophages were found in 96.9% of the *Salmonella* genomes, with more than two intact prophages found within 59.3% of isolates. Interestingly, prophage sequences are present in more than half of the plasmids (51.2%) (Fig. S3b).

Prophages were sorted according to the source of isolation when they were first reported. Indeed, 69.5% were isolated from *Enterobacteriaceae* (Fig. S3c), of which 45.9% belonged to *Salmonella*. Interestingly, most prophages were also reported in other bacterial genera (Fig. S3d), suggesting prophages actively spread among genetically distinct bacterial populations that potentially cohabitate in similar ecological niches.

**Fig 1:**
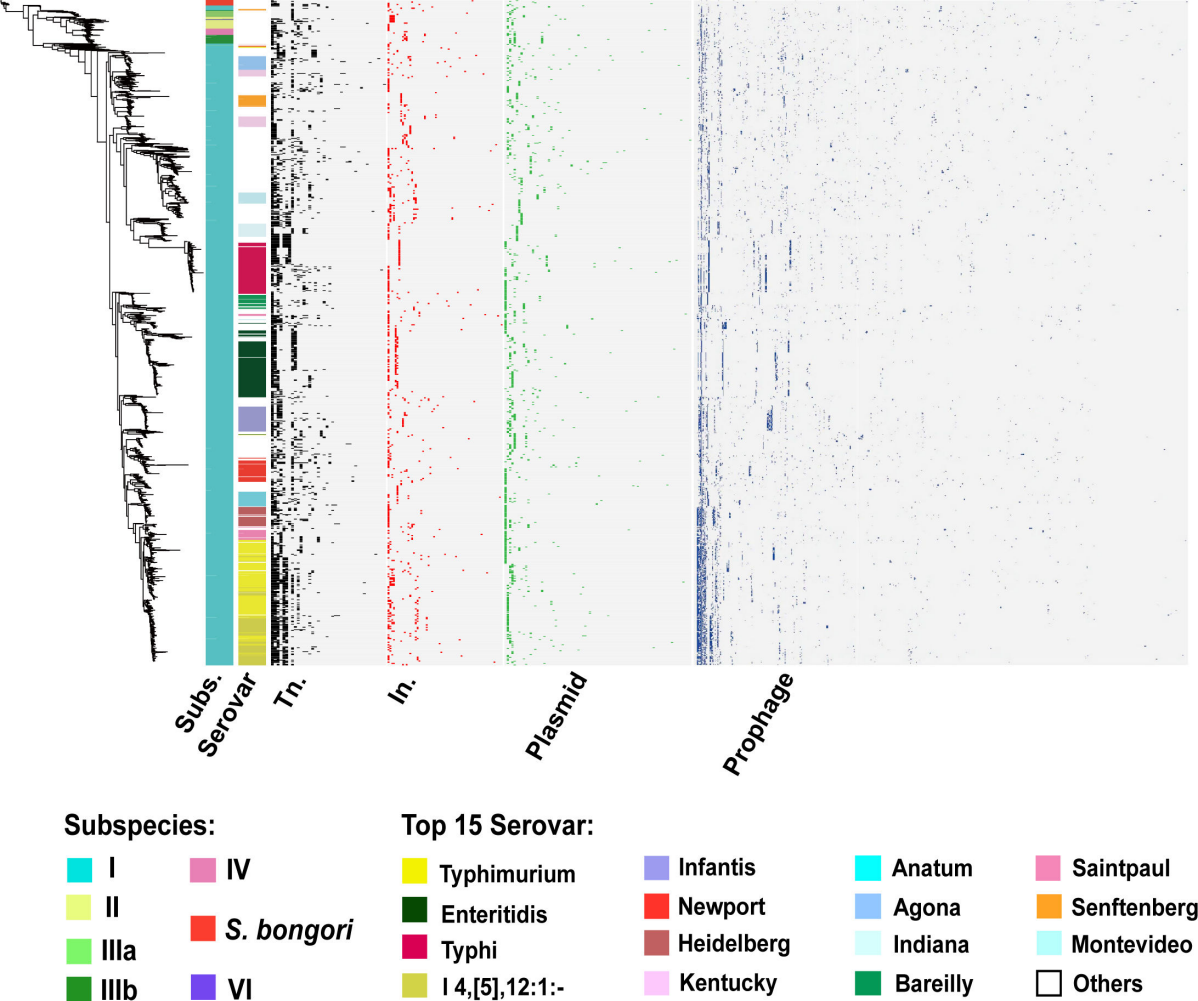
The carriage of four dominant MGEs in *Salmonella* subgroups. The phylogenetic tree was constructed using the pan-genome, and the presence of transposons (Tn.), integrons (In.), plasmids, and prophages was marked by black, red, green, and blue blocks, respectively.

The diversity of integrons and transposons is relatively lower in *Salmonella*. A total of 63 types of integrons and 40 types of transposons were identified. Transposons were found to be more abundant, with 7,348 transposons present in 849 *Salmonella* strains. Among them, Tn6292 (*n* = 3,991) is the most prevalent, followed by Tn21 (*n* = 813), Tn602 (*n* = 623), Tn6205 (*n* = 464), and Tn2012 (*n* = 234). In contrast, only 536 strains carried a total of 826 integrons, with the most frequent integrons belonging to class 1: In498 (*n* = 254), In1469 (*n* = 70), In1368 (*n* = 42), In718 (*n* = 40), and In2 (*n* = 40) (Table S2). Four types of MGEs were exhibited in a serovar-preferential distribution (Fig. 1).

### Plasmid is the predominant reservoir of ARGs

Next, a total of 125 types of ARGs were identified among the 1,817 closed *Salmonella* genome sequences. Compared with other pathways, plasmids demonstrated a significant capacity to carry ARGs (Fig. S4). We observed that 121 types of ARGs could be found on the plasmids, which could be further classified into 12 categories: beta-lactams (*n* = 36) and aminoglycosides (*n* = 31) were predominant, followed by trimethoprim (*n* = 12), macrolides (*n* = 9), quinolones (*n* = 9), tetracyclines (*n* = 6), phenicols (*n* = 5), colistin (*n* = 6), sulfonamides (*n* = 3), multifunctional (*n* = 2), rifampicin (*n* = 1), and fosfomycin (*n* = 1) (Fig. S5a).

**Fig 2:**
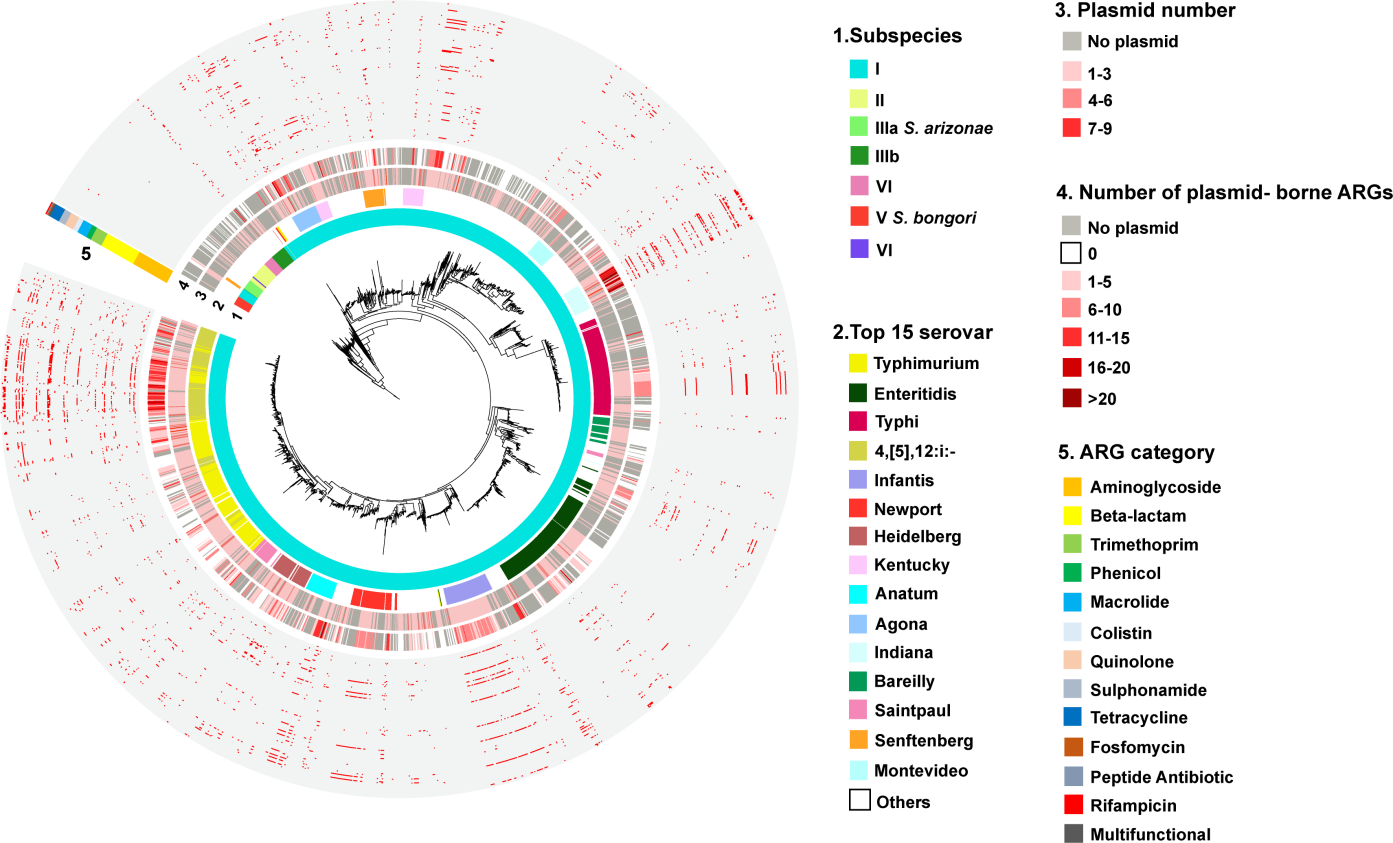
Distribution of plasmids carrying ARGs among *Salmonella* subgroups. The first ring of the tree represents the subspecies of each strain, while the second ring shows the different serovars of *Salmonella*. The third ring displays the number of *Salmonella*-carrying plasmids, and the fourth ring indicates the number of plasmid-bearing ARGs. The fifth ring classifies the ARGs carried on plasmids and is color-coded to indicate the presence (in red) or absence (in light gray) of ARGs.

The variation in plasmid capacity to transfer ARGs was illustrated among different *Salmonella* subgroups (Fig. 2). The results showed that the prevalence of plasmids carrying ARGs was highest in *Salmonella* subspecies I, followed by *Salmonella* subspecies IIIb. Furthermore, we observed that plasmids carrying ARGs tend to cluster at eight specific serovars: 4,[5],12: i:-, Heidelberg, Typhi, Newport, Indiana, Agona, Goldcoast, and Infantis, with nine predominant plasmid types (Fig. S6a). Among them, “*IncHI2, IncHI2A*”- and “*IncA/C2*”-type plasmids that carry T4SS and can self-transfer play a dominant role (Fig. S6b). Interestingly, these plasmids are also present in a serovar-preferential manner, as “*IncHI2, IncHI2A*”-type plasmids tend to be carried by 4,[5],12: i:-, but “*IncA/C2*”-type plasmids are more commonly found in Newport.

### Particular prophages have significant capabilities in ARGs’ dissemination

An unexpectedly diverse set of 101 types of ARGs carried by prophages was identified. However, ARGs belonging to beta-lactams, aminoglycosides, macrolides, and colistin were reduced compared to those maintained by plasmids (Fig. S5b). The distribution of prophages carrying ARGs among different serovars shows a similar pattern to plasmids, highlighting the potential link between plasmids and prophages (Fig. S7). Further analysis showed that prophage “118970_sal3” had the highest prevalence among key serovars (Fig. S8), but ARGs were rarely detected on them. On the contrary, “RCS47” and “SJ46” caught our attention due to their significant ability to carry multiple types of ARGs, suggesting that “RCS47” and “SJ46,” which have been reported in *Escherichia coli*, also play an essential role in acquiring AMR in *Salmonella*. We observed an abundance of ISs and transposases in the structures of “SJ46” and “RCS47,” which potential responsibility for the acquisition of resistance genes (Fig. S9).

### Concordance of transposons and integrons in a serovar manner

Integrons and transposons can transfer ARGs either independently or with the help of other MGEs, such as salmonella genomic island (SGI). Although their relationship is complex, we have found that transposons are more prevalent among *Salmonella* and contain more ARGs than integrons (Fig. S5c and d). Integrons carried by ARGs commonly cluster in Typhimurium and 4,[5],12: i:- (Fig. S10). In1469, which takes aminoglycosides (*aac(6')-Ib*), beta-lactams (*bla*_OXA-1_), and phenicol (*catB3*), is a crucial mediator. Hotspots caused by transposons exhibit greater diversity, and analysis shows that Tn6029 and Tn6205 play vital roles, facilitating the spread of ARGs, especially *aph(6)-Id* and *aph(3'')-Ib*.

*Salmonella* genomic island is an IME derived from *Salmonella enterica* and known for its ability to confer multidrug resistance to *Salmonella* (22). In this study, SGI-1 and SGI-1 variants were found among 25 strains, including serovars 4,[5],12: i:- (*n* = 2), Typhimurium (*n* = 22), and Derby (*n* = 1). SGI-4 was found among 135 strains including serovars 4,[5],12: i:- (*n* = 105) and Typhimurium (*n* = 30). All the SGI sequences were located on chromosomes. Remarkably, we observed the same five categories of resistance genes: *aadA2*, *bla*_CARB-2_, *floR*, *sul1*, and *tet(G*), in all SGI-1 and SGI-1 variants sequences (Fig. S11). In contrast, no resistance genes were detected in SGI-4. Additionally, In498, a class 1 integron, was found in all SGI-1 and SGI-1 variants sequences, which concurs with previous studies (23). Finally, statistics on the source of strains carrying SGI showed that these strains were mainly derived from human and poultry hosts.

Furthermore, we also determined if any transposons carry T4SS and are located on chromosomes (conjugative transposons, ICE). Unfortunately, such elements were not detected. A possible explanation for this is that the transposon carried by *Salmonella* (typically less than 10 kb) is considerably shorter than that held by Gram-positive bacteria and cannot, therefore, take a complete T4SS.

### Plasmid-mediated ARGs increase dramatically

**Fig 3:**
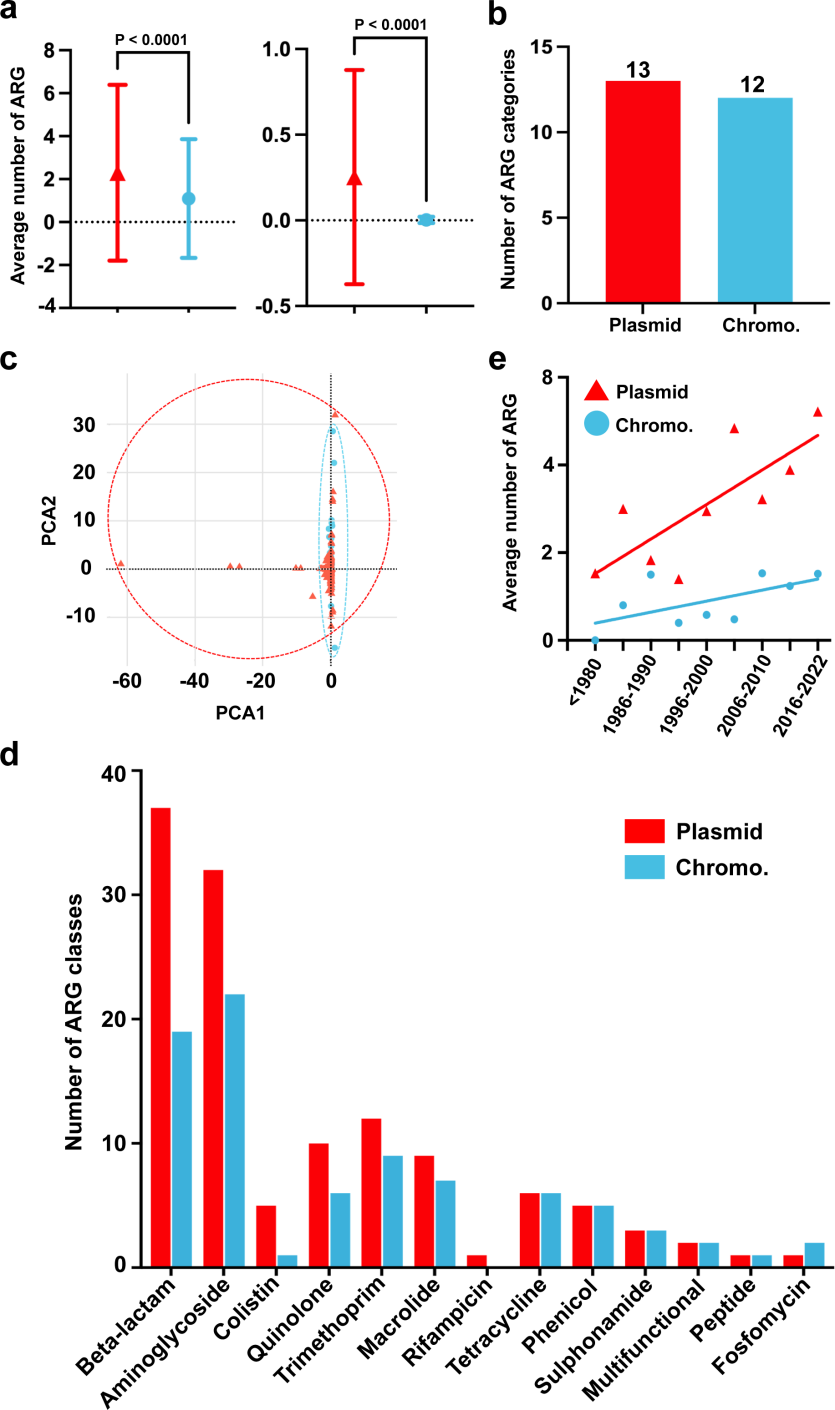
Prevalence of ARGs on plasmids and chromosomes (Chromo.) among 1,817 *Salmonella*. Plasmids are represented in red, while chromosomes are represented in blue. (**a**) The left graph displays the average number of ARGs found on 1,999 plasmids and 1,817 chromosomes. The graph on the right side illustrates the average number of ARGs per 10 kb base length of both plasmids and chromosomes. (**b**) Diversity of ARG category carried by chromosomes and plasmids. (**c**) Principal component analysis (PCA) of ARG types and their abundances on chromosomes and plasmids. (**d**) Specific differences in the categories of ARGs carried by chromosomes and plasmids. On the graph, the vertical axis represents distinct ARG categories, while the horizontal axis denotes the count of distinct ARG classes in each respective category. (**e**) Temporal dynamics of the average number of ARGs on plasmids and chromosomes.

We evaluated the diversity and abundance of ARGs carried by plasmids and chromosomes, respectively. Our results indicate that there are 12 categories of ARGs carried by chromosomes (Fig. 3b). Plasmids carry a higher number of ARGs, particularly in certain categories of ARGs, such as beta-lactam, aminoglycoside, colistin, quinolone, trimethoprim, and macrolide, compared to chromosomes (Fig. 3a, c, and d). Notably, ARGs that induce rifampicin resistance were found solely on plasmids. We further calculated the average number of ARGs carried by the 1,817 chromosomes and 1,999 plasmids. A total of 1,988 ARGs were identified in the chromosomes, averaging 1.094 ARGs per chromosome. The 1,999 plasmids carried a total of 4,588 ARGs, averaging 2.295 ARGs per plasmid. Considering the difference in base length between chromosomes and plasmids, we calculated the average number of ARGs per 10 kb region. The results showed that chromosomes had an average of 0.003 ARGs per 10 kb region, while plasmids had an average of 0.253 ARGs per 10 kb region. Statistical analysis using both methods revealed significant differences between the two groups (*P* < 0.0001). Finally, to emphasize this trend over time, we analyzed the average number of ARGs present on *Salmonella* plasmids and chromosomes from 1911 to 2022 (Fig. 3e). The results showed that the number of plasmid-borne ARGs increased more rapidly than those borne on chromosomes.

### Prophage is a primary facilitator for plasmid-acquiring ARGs

**Fig 4:**
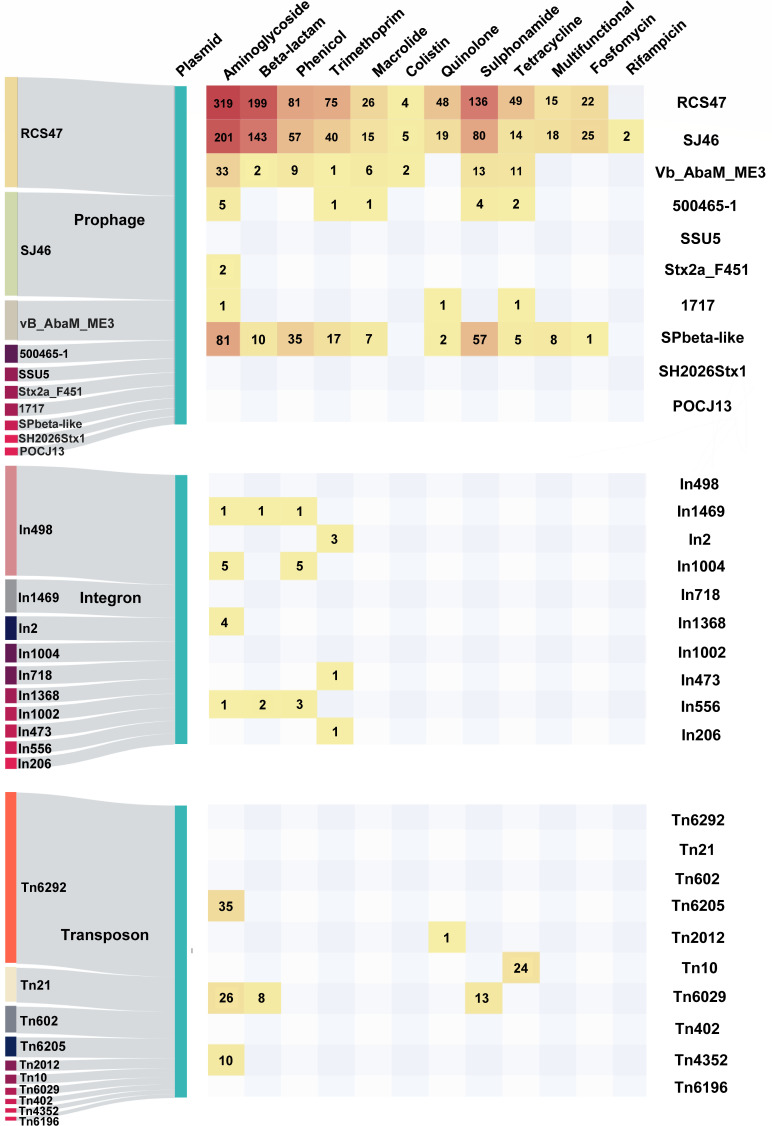
Transposons, integrons, and prophages carried by plasmids and categories of ARGs on them. The Sankey diagrams on the left show the top 10 prophages, integrons, and transposons carried by plasmids, respectively. On the right, the heatmap illustrates the classification of ARGs detected on the top 10 MGEs, with the numbers indicating the specific amount of ARGs carried in that category.

We further identified MGEs located on both plasmids and chromosomes in order to determine their role in the plasmids’ and chromosomes’ plasticity. By utilizing a total of 1,999 closed plasmid sequences, we found that transposons are crucial for plasmid reorganization, followed by prophages and integrons. Specifically, we found a total of 4,740 transposons, 1,669 prophages, and 609 integrons on the plasmids. The most prevalent transposons, prophages, and integrons carried by the plasmids are presented in Fig. 4. However, despite the presence of a significant number of transposons and integrons on the plasmids, no diverse ARGs were associated with them. Instead, we identified a significant abundance and variety of ARGs on plasmid-borne prophages, specifically RCS47, SJ46, and SPbeta-like. The ARGs encompassed a wide range of antibiotics, including aminoglycosides, beta-lactams, phenicol, sulfonamides, and others. This finding suggests that horizontal gene transfer, facilitated by phage invasion, might be a key pathway for the acquisition of ARGs by plasmids.

Then, we investigated the presence of four types of MGEs—transposons, prophages, SGI, and integrons—within the *Salmonella* chromosome and analyzed the ARGs carried by each type of MGEs. Specifically, we detected 16,006 prophages on 1,817 (100%) *Salmonella* chromosomes, 160 SGI sequences on 160 (8.8%) *Salmonella* chromosomes, 2,608 transposon sequences on 473 (26.0%) *Salmonella* chromosomes, and 217 integron sequences on 163 (8.9%) *Salmonella* chromosomes. Interestingly, despite the abundance of prophages on the *Salmonella* chromosome, the number of ARGs it carries is limited (Fig. S12). Further analysis indicated that transposons may serve as the primary reservoir of ARGs on the *Salmonella* chromosome and play a critical role in the vertical transmission of *Salmonella* ARGs.

### Plasmid and prophage-borne critical ARGs dissemination

**Fig 5:**
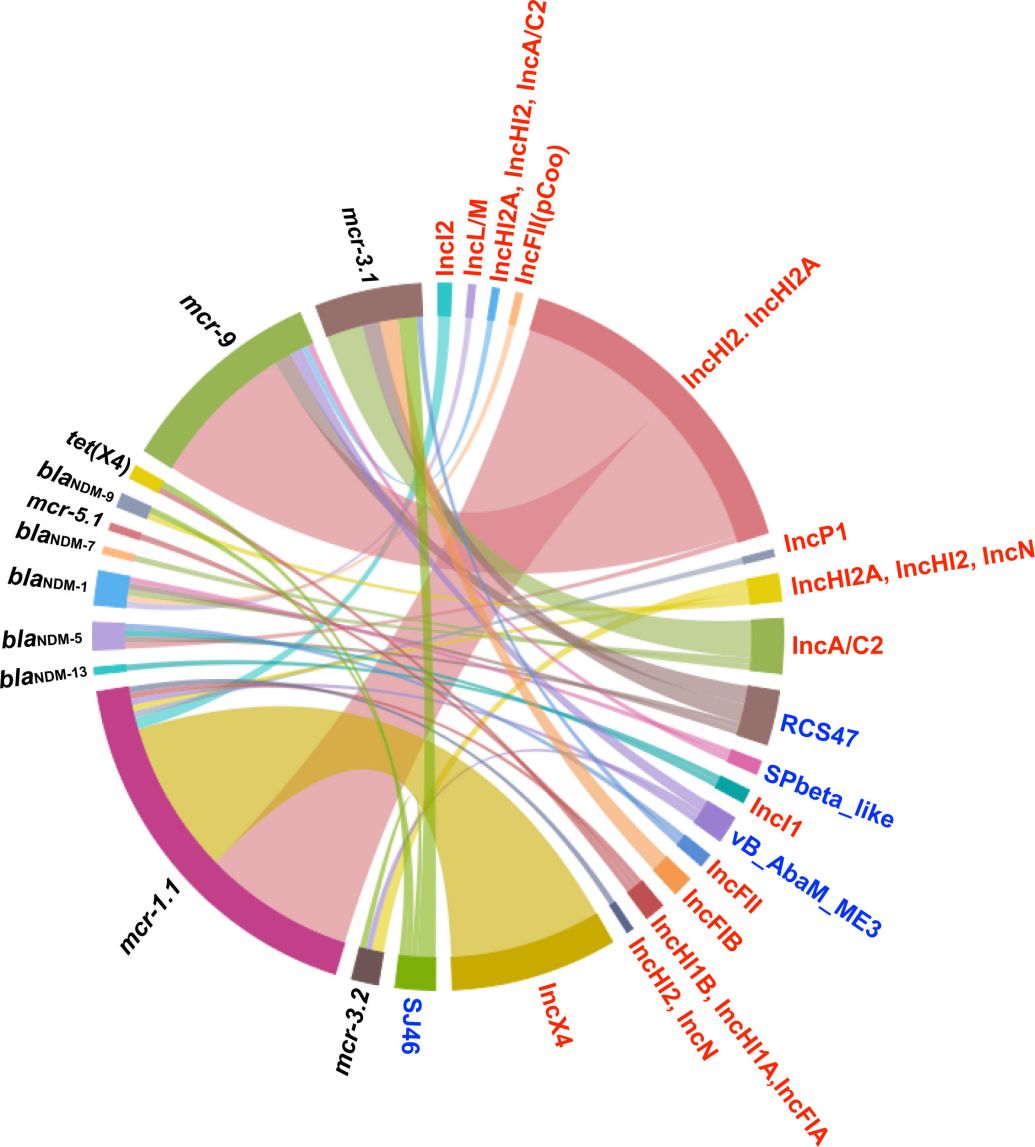
The primary source of *bla*_NDM_, *mcr,* and *tet*(X) family ARGs. The presence of ARGs, plasmids, and prophages is marked in black, red, and blue fonts, respectively.

The *bla*_NDM_, *mcr*, and *tet*(X) ARG families are of great interest, as they represent key ARGs that can lead to bacterial resistance to last-resort antibiotics. In our study, we identified a total of 114 key ARGs among 113 plasmids and 20 key ARGs on 20 prophages (Table S3). Among these, the *mcr* family ARGs are the most abundant, specifically *mcr-1.1* and *mcr-9*, which are predominantly present on “*IncHI2, IncHI2A*”- and “*IncX4*”-type plasmids (Fig. 5). Notably, plasmid “AP023312.1” contains both *mcr-5.1* and *mcr-1.1*, which carry three replicons: “*IncHI1A*,” “*IncFIA*,” and “*IncHI1B*.” Meanwhile, the *bla*_NDM_ and *tet*(X) family ARGs are relatively rare and found only on 11 plasmids and 5 prophages; *IncA/C2*-type plasmids and prophage RCS47 are the primary carriers.

It has been reported that specific ISs associated with *bla*_NDM_ and *mcr* family ARGs can mobilize these genes within *E. coli* by utilizing transposons (24–26). We observed a similar regularity in *Salmonella*. For *mcr* family ARGs, IS*Kpn40* was present near all *mcr-3* genes, while a strong association between IS*30* and *mcr-1.1* was noted (Fig. S13). Also, three types of IS genes were present near the *bla*_NDM_ family ARGs: IS*26*, IS*Aba125*, and IS*Sbo1* (Fig. S14).

### Socioeconomic impacts on the prevalence of MGE-borne ARGs

**Fig 6:**
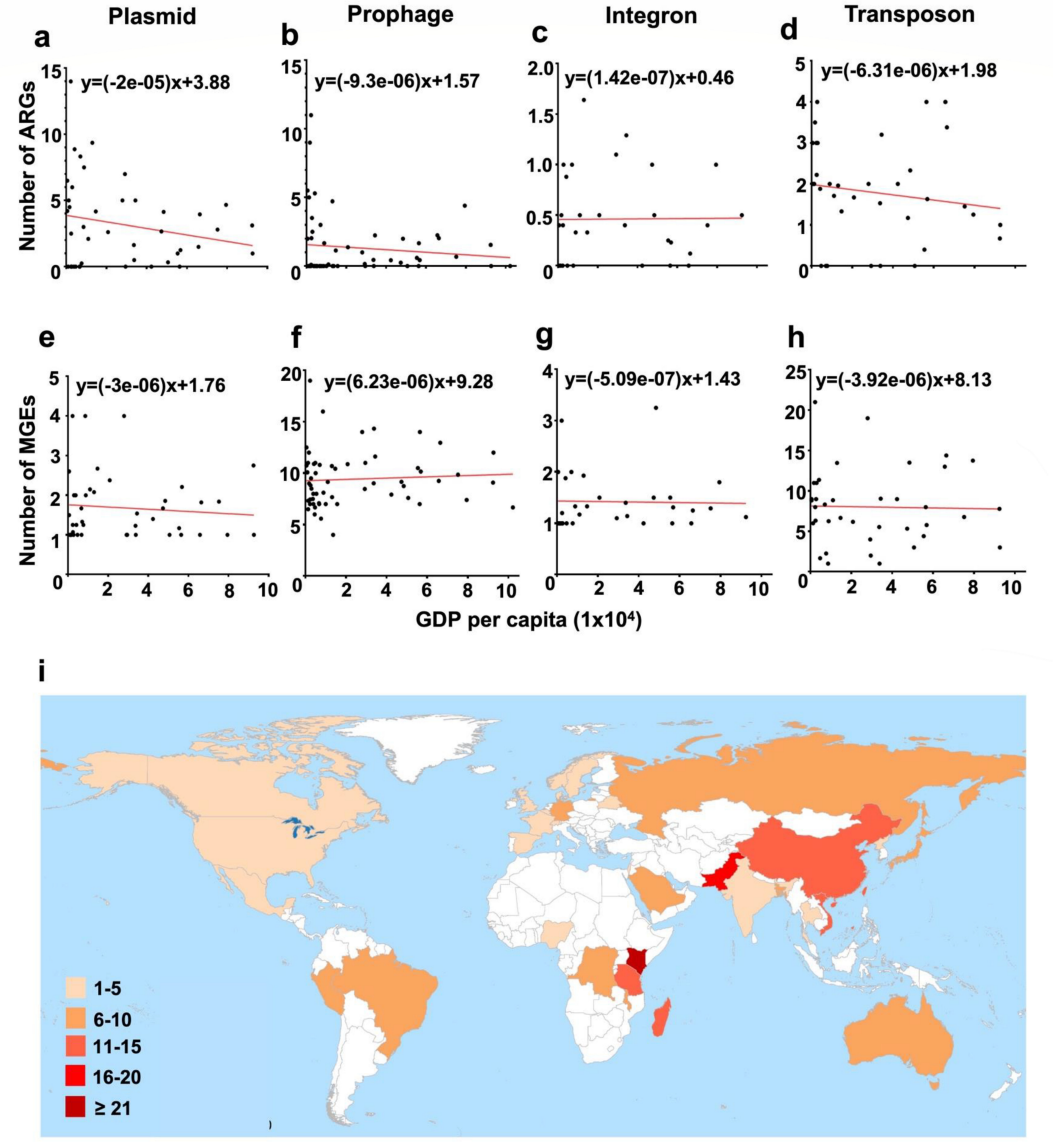
An association analysis of MGE-carrying *Salmonella* with GDP per capita. A ranking in order of MGE type (plasmid, prophage, integron, and transposon). (**a–d**) Trend analysis of GDP per capita and the average number of MGEs carried by *Salmonella*. (**e–h**) Trend analysis of GDP per capita and the average number of ARGs carried by MGEs. (**i**) Average number of ARGs carried by MGEs in different countries.

We compared *Salmonella* isolated from countries with different levels of GDP per capita and found that *Salmonella* from higher-income countries carried fewer integrons, transposons, and plasmids (Fig. 6a to d). This general trend was also evident in the frequency of MGE-borne ARGs (Fig. 6e to h). Economically developed regions like Europe and the Americas had fewer MGE-bearing ARGs than African and Asian regions (Fig. 6i).

Further, we compared the MGEs and MGE-borne ARGs carried by *Salmonella* isolated from human fecal and blood sources. Using a pretrained invasiveness index model, we found that *Salmonella* isolated from blood samples had a significantly higher invasive ability than those isolated from fecal samples (Fig. S15). However, the total number of MGEs and MGE-bearing ARGs was not significantly different between the two groups. This suggests that the invasiveness of *Salmonella* in humans may not be strongly related to the MGEs analyzed in our study. Finally, a comparison of animal, human, and environmental *Salmonella* genomes revealed that human and animal isolates typically carried more MGEs and MGE-borne ARGs than those isolated from environmental sources (Fig. S16).

## DISCUSSION

The emergence of AMR is a critical public health concern. Horizontal transfer of MGEs, as key drivers, facilitates the acquisition of ARGs in bacteria (27). Nevertheless, the specific contribution of distinct MGEs is poorly understood. Here, four dominant MGEs and their associated MGEs in *Salmonella* were investigated. Only closed genome data were collected to eliminate the possible effects of incomplete splicing on the results. Our analyses show that plasmid is the largest ARG reservoir in *Salmonella*, representing the most diverse and abundant. However, prophages were identified as the second largest ARG reservoir, exhibiting a similar pattern of ARG distribution as plasmids. Only rifampicin was not detected on prophage sequences. On the other hand, although integrons, transposons, and SGI were frequently reported in *Salmonella*, they carried fewer ARGs than plasmids and prophages.

By phylogeny analysis, we observed a divergence in the distribution of MGEs and MGE-carrying ARGs among different *Salmonella* subgroups. Subspecies I of *Salmonella* harbored significantly more MGEs and MGE-carrying ARGs. Specific serovars, such as 4,[5],12: i:-, Indiana, Infantis, and Typhimurium, which have been widely reported in recent years (28), observed a strong MGE-ARG link. Our findings further indicate that the higher levels of AMR observed in these serovars may be due to the prevalence of critical MGEs, including “*IncHI2*”-, *“IncHI2A*”-, and “*IncA/C2*”-type plasmids, as well as prophages RCS47 and SJ46. Interestingly, *Salmonella* IIIb, commonly associated with sheep (29), also presents a high percentage of ARGs carriage. This finding is consistent with our results that *Salmonella* from livestock and poultry sources is more likely to carry MGEs and ARGs.

To investigate the potential origin of plasmids carrying ARGs, we compared the presence of transposons, integrons, and prophages located on plasmids and carrying ARGs. The results revealed a significant number of ARGs on plasmids also located on prophages, specifically RCS47, SJ46, and SPbeta-like. These multihost prophage sequences have also been reported in *E. coli* and *Bacillus* (30). Thus, we speculate that the widespread distribution and invasion of ARG-borne phages could be plasmids' primary pathway to acquiring ARGs. Notably, ARGs on plasmids increased rapidly from 1911 to 2022, suggesting that ARG transfer via this pathway is becoming more common.

The dissemination of the *bla*_NDM_, *mcr*, and *tet*(X) family of ARGs has been mainly considered through plasmids; however, our research has shown that these critical ARGs are also present on plasmid-carrying prophages, particularly among *Salmonella* strains isolated from China and the USA. This discovery offers a novel perspective on transmitting these key ARGs in *Salmonella*. For instance, *tet*(X4), previously identified on plasmid (31), was also located on the prophage SJ46 in our study, which had not been reported before. Interestingly, the spread of these key ARGs is also related to transposon structures such as IS30 and ISKpn40. Previous studies reported a similar structure in *E. coli*, and our study observed a similar pattern in *Salmonella*, highlighting the complexity and cross-species nature of these critical ARGs in the dissemination process.

It has been reported that the correlation between the number of ARGs carried by bacteria and economic development level is negative (32). MGE-bearing ARGs show the same pattern, implicating that the policy to combat AMR is effective, while the low-income regions are still the major areas for the dissemination of antibiotic-resistant *Salmonella*, and aggressive promotion of resistance reduction policies in these regions is necessary. Also, it is interesting that the rate of decline is variable for different types of MGE-carrying ARG. We found that with higher GDP per capita, ARGs carried by plasmids show the strongest negative correlation, followed by prophages and transposons. However, a surprising positive correlation was observed between the number of ARGs carried by integrons and the GDP per capita (Fig. 6c). It is speculated that the advancement of medical care in high-income regions has hindered the transmission of ARGs among *Salmonella*, also resulting in a decrease in the number of ARGs carried by MGEs. Integrons might possess distinct transmission mechanisms, resulting in diverse developmental trends; thus, further tailored policies to combat AMR are needed. Moreover, the current surveillance of ARGs prevalence primarily emphasizes plasmid analysis, while our study indicates that the role played by phages is underestimated. Monitoring the spread of phages should also be considered in future investigations. Finally, the distribution of ARGs for *Salmonella* in ecological niches tends to vary. *Salmonella* originating from animal sectors with the highest rates of ARGs. It is crucial to develop preventive measures specifically designed for the farming industry, such as mitigation of zoonotic transmission during the farming process and improving food hygiene, therefore, against increasing rates of AMR *Salmonella*.

The current study also has limitations. The first limitation would be the data bias. Given the access and availability of long-read technologies, there are more data produced from high-income countries. Likewise, widely recognized *Salmonella* serotypes such as Typhimurium are frequently detected in clinical cases, potentially introducing sample bias. Additionally, the data set utilized in this study exhibited a higher detection rate for the *mcr* and *bla*_NDM_ family of ARGs. This could be attributed to the tendency that bacteria with critical antibiotic resistance are more likely to be subjected to WGS, in particular for the closed genome, as indicated in this study. Secondly, the detection of MGEs and ARGs highly depends on sophisticated databases that are under the updating process. The current method for such estimations may underestimate the exact prevalence of MGEs and ARGs. Nevertheless, by using the largest closed genomes from a single bacterial genus *Salmonella*, we pinpoint the mobilome is highly associated with serovar-based evolution, leading to the distinct *Salmonella* resistome. Further socioeconomic correlation studies highlighted distinct resistome and mobilome burdens between high- and low-income settings. The knowledge gained in this study may guide updated policies to slow down AMR dissemination.

## MATERIALS AND METHODS

### Database assembly

We screened and selected 1,816 complete genome sequences of *Salmonella* and downloaded these genome files from GenBank (accessed on 10 September 2022), which were isolated between 1911 and 2022 (Table S1). Additional information about *Salmonella* strains was also collected, including the collection time, serovar distribution, and source of isolation. A laboratory-derived *Salmonella* (R51) was also included in this analysis (33, 34 ), which was conducted by the Pacbio RSII sequencing (Beijing Novogene Co. Ltd).

### Salmonella subspecies and serovar determination

All assemblies, including publicly available data, were quality-checked using FastQC v0.11.9 and performed serovar predictions using SISTR v1.1 (35) and Seqsero2 (36) to improve the accuracy of serovar information (37, 38 ). Here, we integrated the serovar information provided by NCBI with the error of software prediction to estimate the most likely serovar of each *Salmonella*. The determination of *Salmonella* subspecies followed the ninth edition of the classification manual formulated by World Health Organization (WHO) (39).

### Plasmid sequence recovery

Biopython v1.8 was used to recover plasmid sequences into single files in “.fasta” format. To facilitate the subsequent analysis, the database containing 1,817 *Salmonella* complete WGS data was artificially partitioned into two data sets: the plasmid-only data and the chromosome-only data. In this study, each plasmid sequence has a unique and quarriable ID.

Plasmid file size (kb), base length (bp), and GC content (%) were calculated by localized Python3 scripts. “os.path.getsize()” function was used to calculate the plasmid file size (kb), the “len()” function was used to calculate the plasmid base length(bp), and the “count()” function was used to calculate the plasmid GC content (%).

### Detection of prophages in *Salmonella* genomes

The prophages were detected in *Salmonella* genomes using Phaster tool (40). The genomic data were split into two temporary databases based on the number of contigs; one data set containing single contig files and the other containing multiple contig files. The two databases were imported into the Phaster pipeline separately with default parameters. The prophages were automatically classified into three groups based on the matched DNA sequences (coding sequences [CDSs]): intact, questionable, and incomplete. In the current study, questionable and incomplete prophages are considered defective prophages.

### Detection of integrons and transposons

BacAnt (13) tool was used to detect integrons and transposons in the genomes carried by *Salmonella*. The databases used by BacAnt include NCBI, INTEGRALL (41), and THE TRANSPOSON REGISTRY (42). The BacAnt workflow was implemented on a localized server running Cent OS. Only integrons or transposons with a similarity greater than 60% and a coverage greater than 60% were identified. The identified integron or transposon sequences are separated by Python3 scripts into multi-fasta files for future identification of the carrying ARGs.

### Phylogenomic relationship within *Salmonella* serovars

The phylogenetic tree of *Salmonella* was constructed based on the pan-genome analysis. Genome annotation was first performed using Prokka v1.14.6 (43). The annotation files (“GFF3” format) were imported into Roary v3.13.0 (44) for pan-genome analysis. IQ-TREE v1.6.12 (45) was used to generate the 01 profile matrix as a maximum-likelihood phylogenetic tree (1,000 bootstraps) using the model TVM + F + ASC + R3. Phylogenetic trees were displayed and annotated using ITOL (46).

### *In silico* analysis of MGEs sequences

The plasmid replicon types and SGI-1 were identified using Abricate v1.0.1 software with the PlasmidFinder database (47) and local SGI Blastn index database. Only sequences with similarity greater than 95% and coverage greater than 60% can be determined (48).

A phylogenetic tree based on core single nucleotide polymorphisms (core SNPs) was constructed for the most frequent plasmid type. Core SNPs loci were obtained using Snippy v4.4.4. The phylogenetic tree was then calculated and visualized using iTOL. In addition, BRIG (49) (identify threshold = 50%) was used for comparative analysis of the different plasmid types.

ICEfinder (50) was used to detect the presence of T4SS components on the genomic sequence using default parameters.

The ARGs were detected by Abricate v1.0.1 with the ResFinder (51) database. Only ARGs with similarity greater than 95% and coverage greater than 98% were identified (52). Each identified ARG was manually classed into different antimicrobial categories to which they belong.

## Data Availability

All complete genomic data for *Salmonella* is available at NCBI. Table S1 provides the accession numbers, isolate source, and corresponding estimates of GDP per capita for the countries in 2022. The GenBank accession number of the sequence used to detect the SGI-1 and SGI-1 variants is AF261825.2. Also, the GenBank accession number of the sequence used to detect SGI-4 is MN730129.1. The open-source software used in this study includes Biopython (https://biopython.org), FastQC (https://www.bioinformatics.babraham.ac.uk/projects/fastqc/), BacAnt (https://github.com/xthua/bacant), Abricate (https://github.com/tseemann/abricate), Snippy (https://github.com/tseemann/snippy), Roary (https://github.com/sanger-pathogens/Roary), Prokka (https://github.com/tseemann/prokka), SISTR (https://github.com/phac-nml/sistr_cmd), SeqSero2 (https://github.com/denglab/SeqSero2), ICEfinder (https://bioinfo-mml.sjtu.edu.cn/ICEfinder/index.php), IQtree (https://github.com/iqtree/iqtree2), and Staramr (https://github.com/phac-nml/staramr). Additional code for data processing can be found on GitHub (https://github.com/tjiaa).

## SUPPLEMENTAL MATERIAL

Supplemental Figures msystems.00883-23-s0001.pdf Figures S1-S16.

Supplemental Legends msystems.00883-23-s0002.pdf Legends for supplemental figures and tables.

Table S1 msystems.00883-23-s0003.xlsx Summary of 1,817 complete *Salmonella* genome sequences collected from GenBank and the GDP per capita of the isolating country in 2022.

Table S2 msystems.00883-23-s0004.xlsx Details of the four MGEs (plasmid, prophage, integron, transposon) detected on 1,817 *Salmonella*.

Table S3 msystems.00883-23-s0005.xlsx Information on plasmids, prophages, and serovars of *Salmonella* carrying *bla*_NDM_, *mcr*, and *tet*(X) family ARGs.

Table S4 msystems.00883-23-s0006.xlsx Chromosomes and plasmid-borne ARGs of 1,817 *Salmonella* and the ARGs located on four types of chromosomes carrying MGE.

ASM does not own the copyrights to Supplemental Material that may be linked to, or accessed through, an article. The authors have granted ASM a non-exclusive, world-wide license to publish the Supplemental Material files. Please contact the corresponding author directly for reuse.
