# Supplementary material for: Mobilome-driven partitions of the resistome in Salmonella
Source: mSystems. 2023 Oct 19;8(6):e00883-23. doi: 10.1128/msystems.00883-23 (PMC10734508; doi:10.1128/msystems.00883-23)
Supplement: Supplemental Figures — Figures S1-S16. [file msystems.00883-23-s0001.pdf]

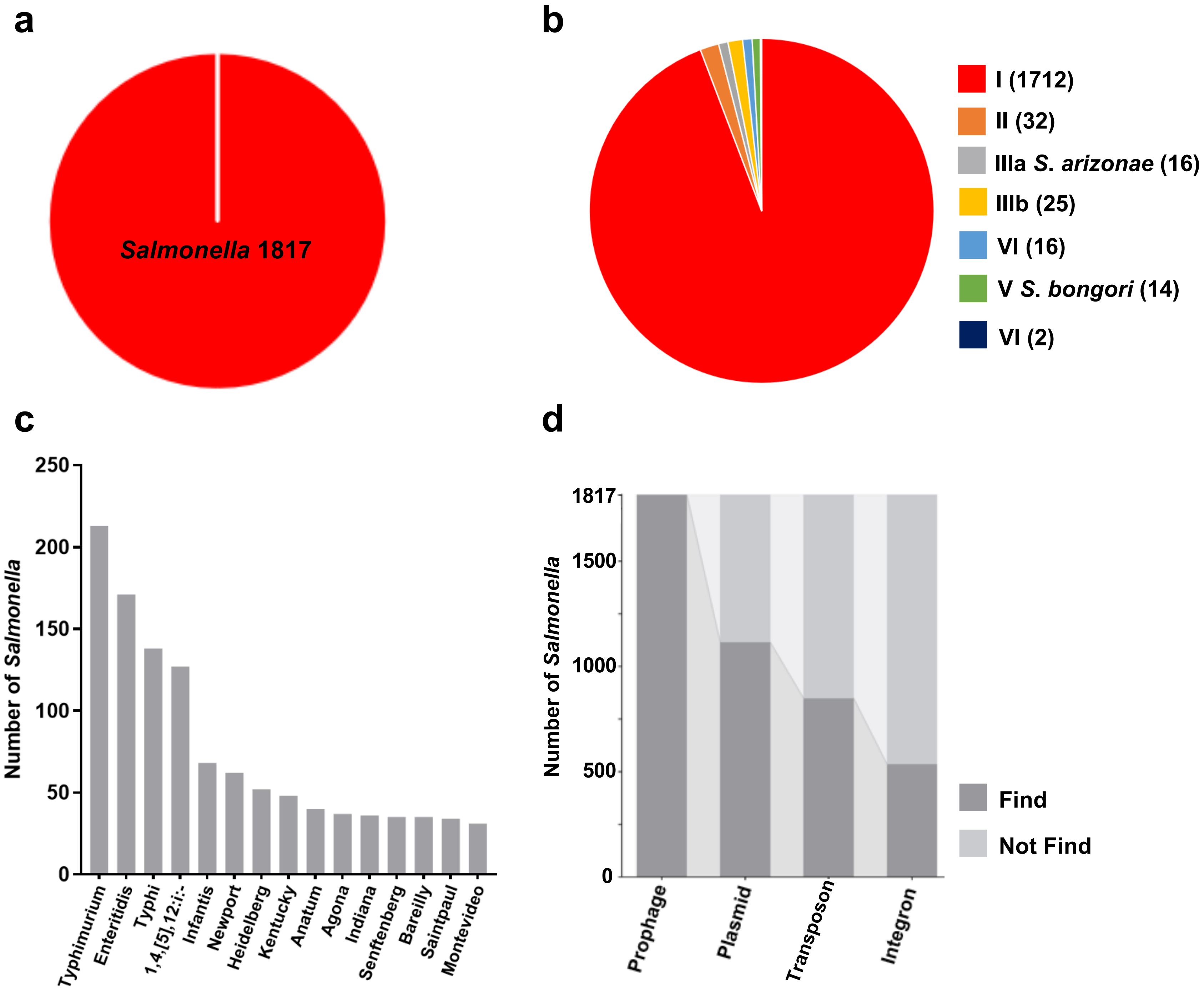

a
Salmonella 1817
b
I (1712)
II (32)
IIIa S. arizonae (16)
IIIb (25)
VI (16)
V S. bongori (14)
VI (2)
c
Number of Salmonella
250
200
150
100
50
0
Typhimurium
Enteritidis
Typhi
1,4,[5],12:i:-
Infantis
Newport
Heidelberg
Kentucky
Anatum
Agona
Indiana
Senftenberg
Bareilly
Saintpaul
Montevideo
d
Number of Salmonella
1817
1500
1000
500
0
Prophage
Plasmid
Transposon
Integron
Find
Not Find

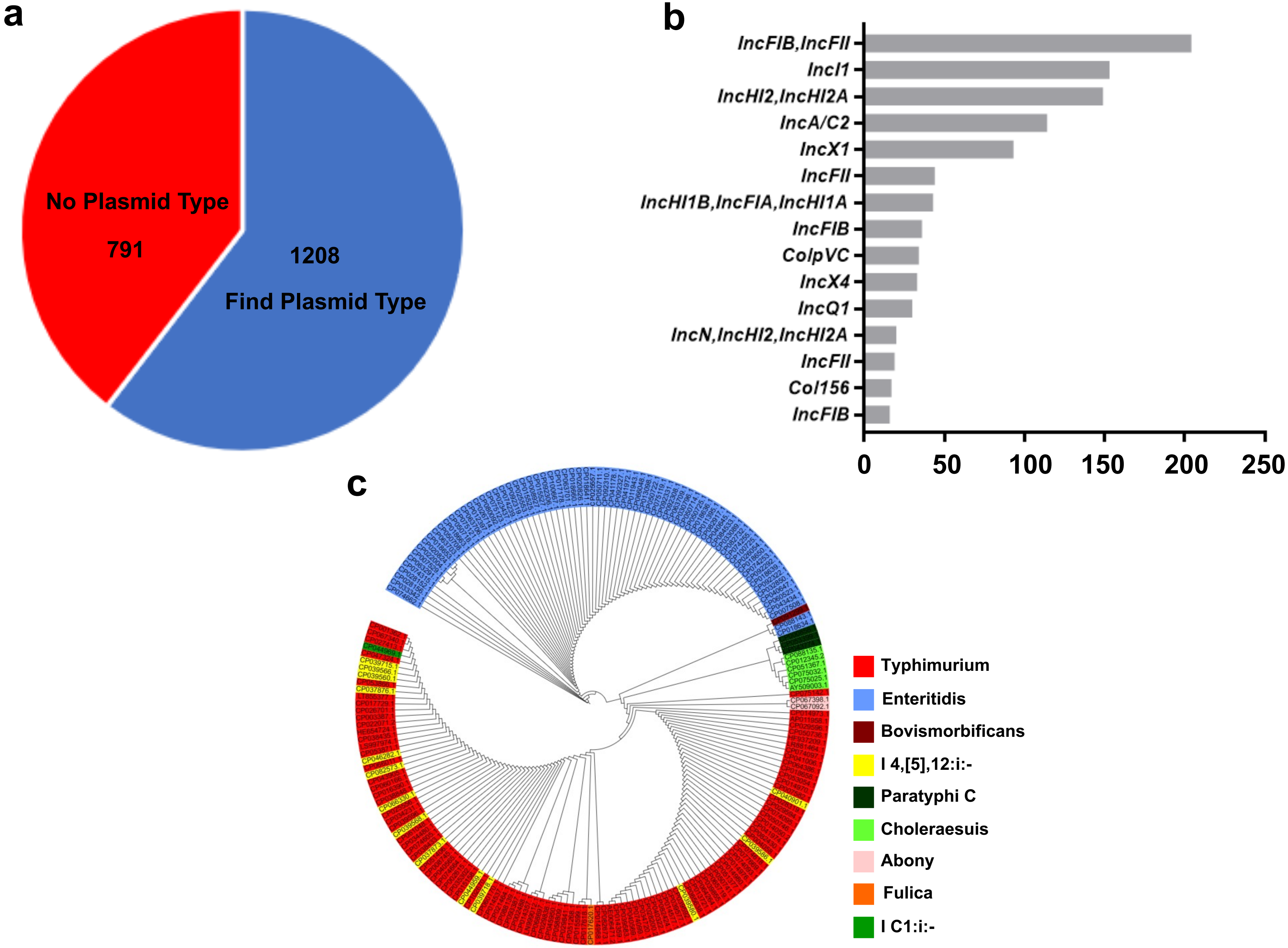
a
No Plasmid Type
791
1208
Find Plasmid Type
b
IncFIB,IncFII
IncI1
IncHI2,IncHI2A
IncA/C2
IncX1
IncFII
IncHI1B,IncFIA,IncHI1A
IncFIB
ColpVC
IncX4
IncQ1
IncN,IncHI2,IncHI2A
IncFII
Col156
IncFIB
0
50
100
150
200
250
c
Typhimurium
Enteritidis
Bovismorbificans
I 4,[5],12:i:-
Paratyphi C
Choleraesuis
Abony
Fulica
I C1:i:-

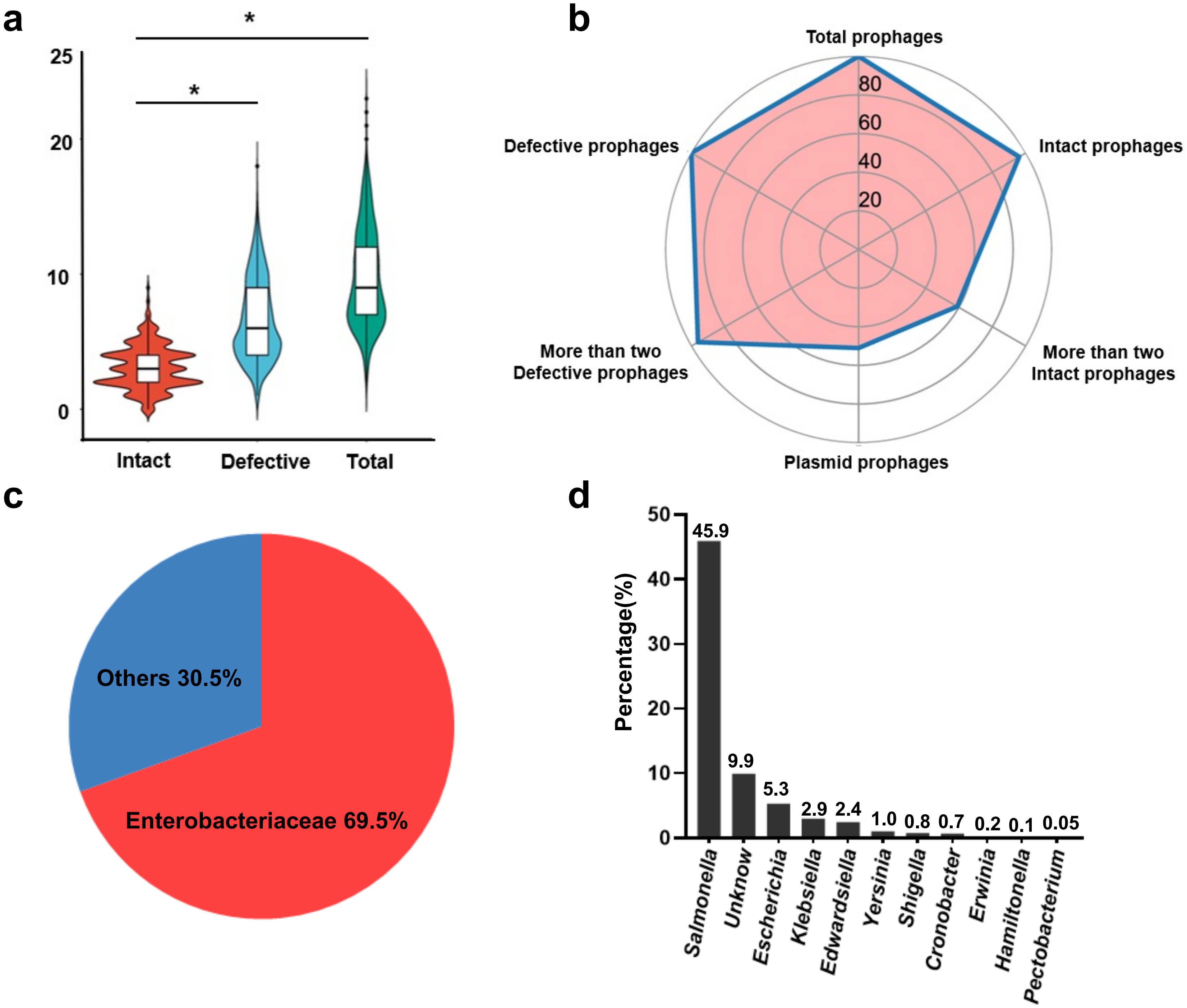

a
*
*
25
20
10
0
Intact
Defective
Total
b
Total prophages
80
60
40
20
Defective prophages
Intact prophages
More than two
Defective prophages
More than two
Intact prophages
Plasmid prophages
c
Others 30.5%
Enterobacteriaceae 69.5%
d
Percentage(%)
50
40
30
20
10
0
45.9
9.9
5.3
2.9
2.4
1.0
0.8
0.7
0.2
0.1
0.05
Salmonella
Unknow
Escherichia
Klebsiella
Edwardsiella
Yersinia
Shigella
Cronobacter
Erwinia
Hamiltonella
Pectobacterium

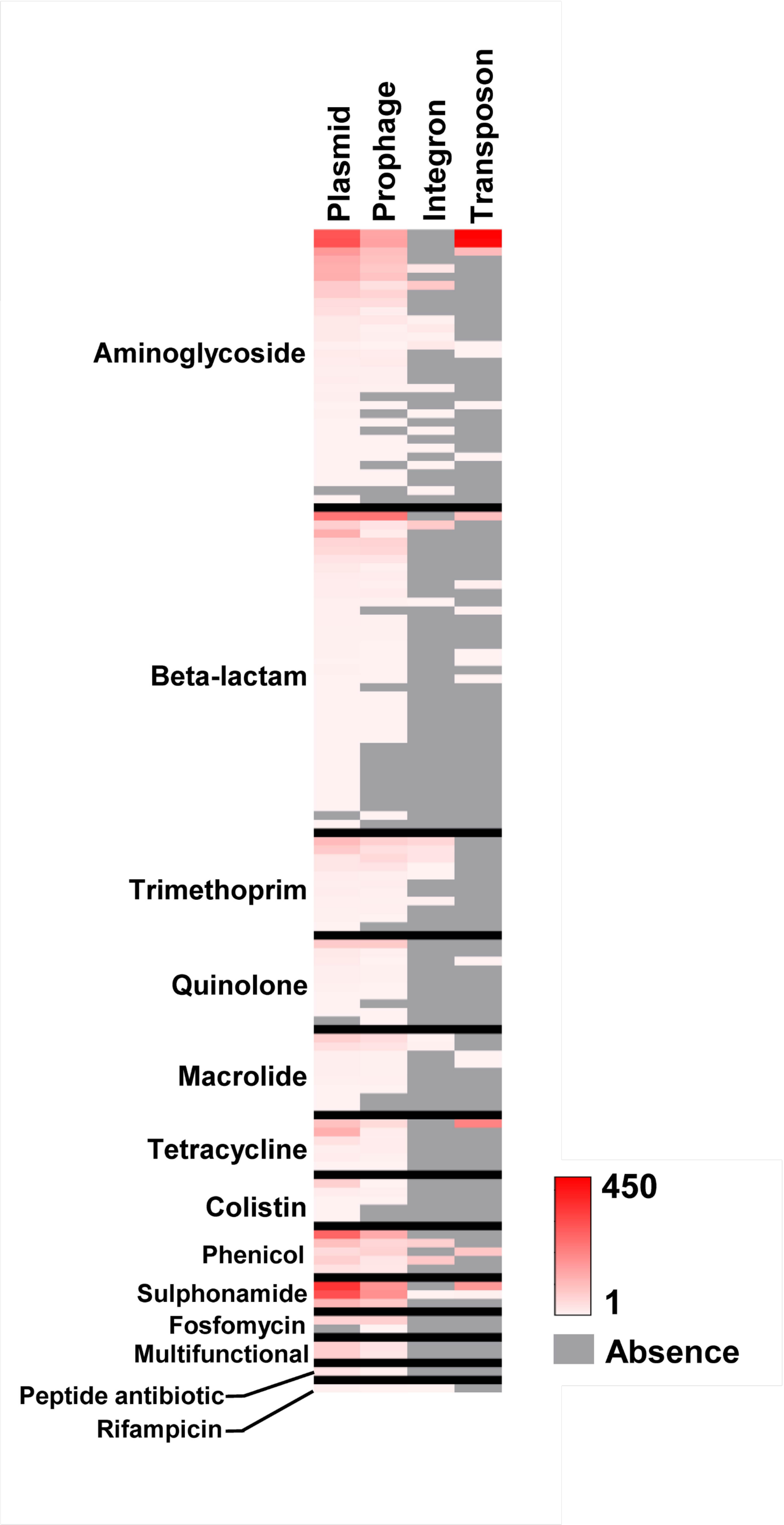

Plasmid
Prophage
Integron
Transposon
Aminoglycoside
Beta-lactam
Trimethoprim
Quinolone
Macrolide
Tetracycline
Colistin
Phenicol
Sulphonamide
Fosfomycin
Multifunctional
Peptide antibiotic
Rifampicin
450
1
Absence

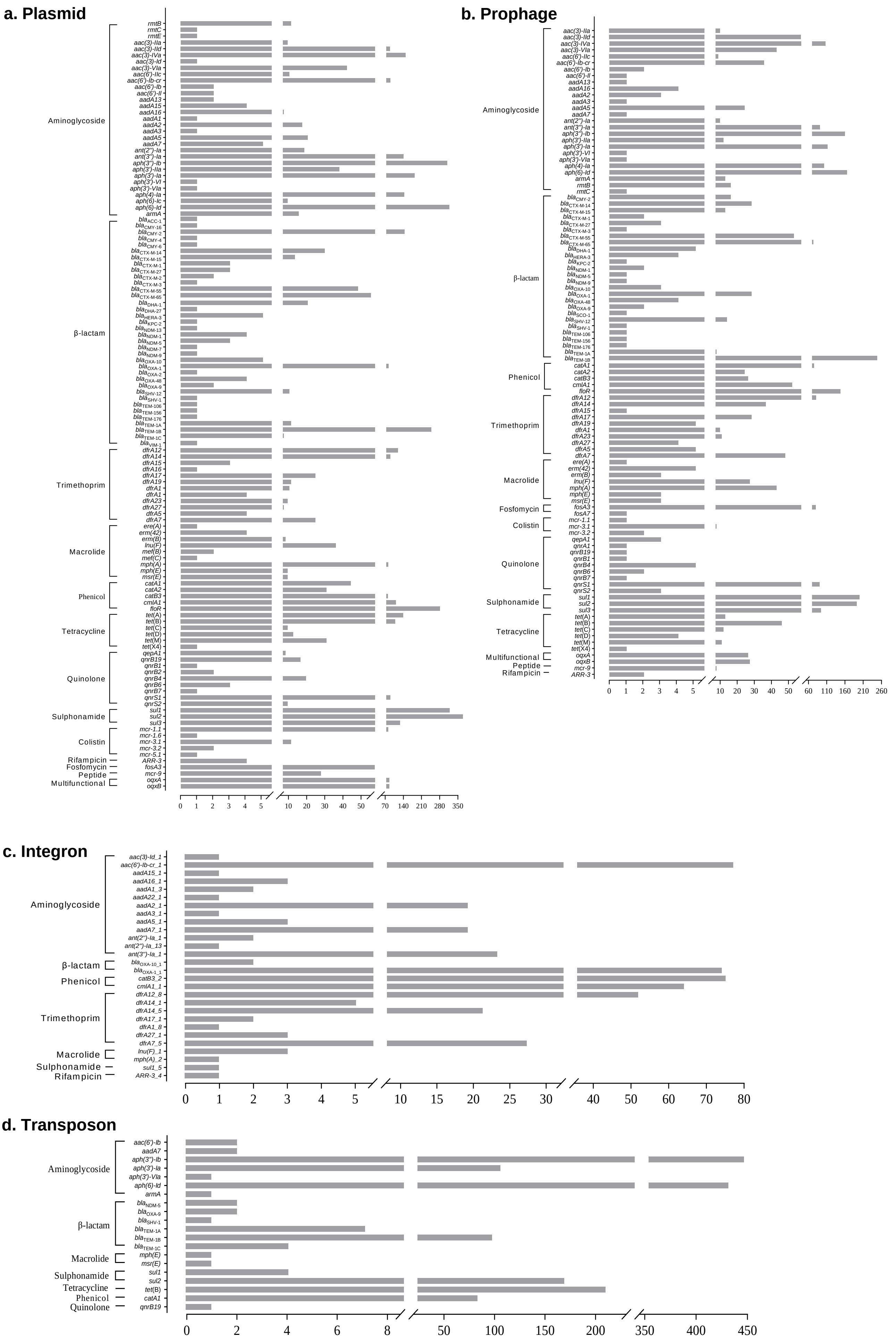

a. Plasmid
Aminoglycoside
β-lactam
Trimethoprim
Macrolide
Phenicol
Tetracycline
Quinolone
Sulphonamide
Colistin
Rifampicin
Fosfomycin
Peptide
Multifunctional
0 1 2 3 4 5 10 20 30 40 50 70 140 210 280 350
b. Prophage
Aminoglycoside
β-lactam
Phenicol
Trimethoprim
Macrolide
Fosfomycin
Colistin
Quinolone
Sulphonamide
Tetracycline
Multifunctional
Peptide
Rifampicin
0 1 2 3 4 5 10 20 30 40 50 60 110 160 210 260
c. Integron
Aminoglycoside
β-lactam
Phenicol
Trimethoprim
Macrolide
Sulphonamide
Rifampicin
aac(3)-Id_1
aac(6')-Ib-cr_1
aadA15_1
aadA16_1
aadA1_3
aadA22_1
aadA2_1
aadA3_1
aadA5_1
aadA7_1
ant(2")-Ia_1
ant(2")-Ia_13
ant(3")-Ia_1
blaOXA-10_1
blaOXA-1_1
catB3_2
cmlA1_1
dfrA12_8
dfrA14_1
dfrA14_5
dfrA17_1
dfrA1_8
dfrA27_1
dfrA7_5
lnu(F)_1
mph(A)_2
sul1_5
ARR-3_4
0 1 2 3 4 5 10 15 20 25 30 40 50 60 70 80
d. Transposon
Aminoglycoside
β-lactam
Macrolide
Sulphonamide
Tetracycline
Phenicol
Quinolone
aac(6')-Ib
aadA7
aph(3")-Ib
aph(3')-Ia
aph(3')-VIa
aph(6)-Id
armA
blaNDM-5
blaOXA-9
blaSHV-1
blaTEM-1A
blaTEM-1B
blaTEM-1C
mph(E)
msr(E)
sul1
sul2
tet(B)
catA1
qnrB19
0 2 4 6 8 50 100 150 200 350 400 450

a.

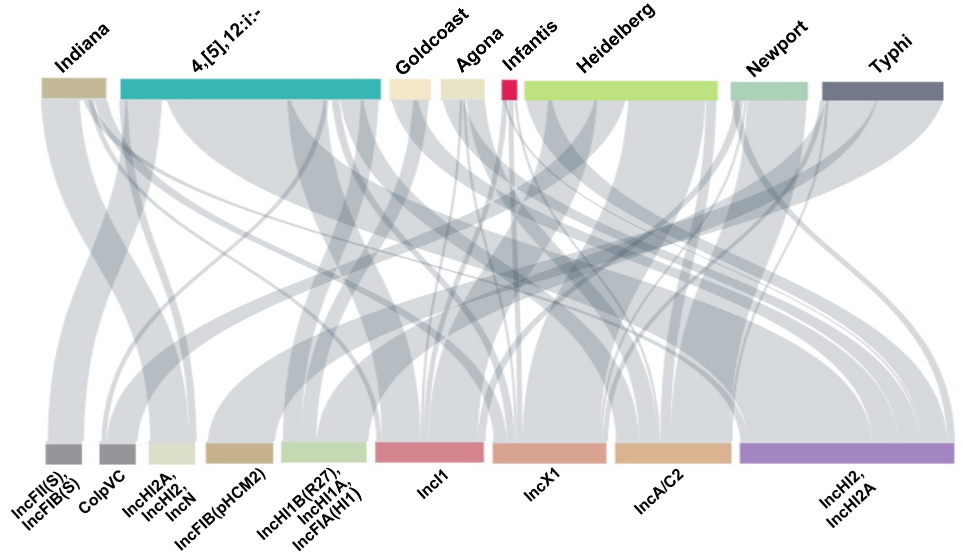


b.

| | Aminoglycoside | Sulphonamide | Beta-lactam | Phenicol | Trimethoprim | Tetracycline | Multifunctional | Macrolide | Quinolone | Colistin | Fosfomycin | Peptide | Rifampicin |
|---|---|---|---|---|---|---|---|---|---|---|---|---|---|
| IncHI2, IncHI2A* | 268 | 163 | 123 | 111 | 56 | 12 | 56 | 36 | 29 | 21 | 15 | 10 | 2 |
| IncA/C2* | 110 | 56 | 68 | 48 | 8 | 42 | | 5 | 6 | 5 | | | |
| IncX1* | 9 | 3 | 6 | 3 | | | 2 | 3 | 1 | | | | |
| IncI1* | 24 | 8 | 28 | 1 | 1 | | | 1 | | | | | |
| IncHI1B(R27), IncHI1A, IncFIA(HI1) | 41 | 54 | 27 | 33 | 27 | 34 | | 1 | 7 | 2 | | | |
| IncFIB(pHCM2) | | | | | | | | | | | | | |
| IncHI2A, IncHI2, IncN* | 95 | 43 | 40 | 35 | 16 | | 20 | 6 | 3 | 2 | 9 | | |
| ColpVC | | | | | | | | | | | | | |
| IncFII(S), IncFIB(S) | | | | | 1 | 1 | | | | | | | |

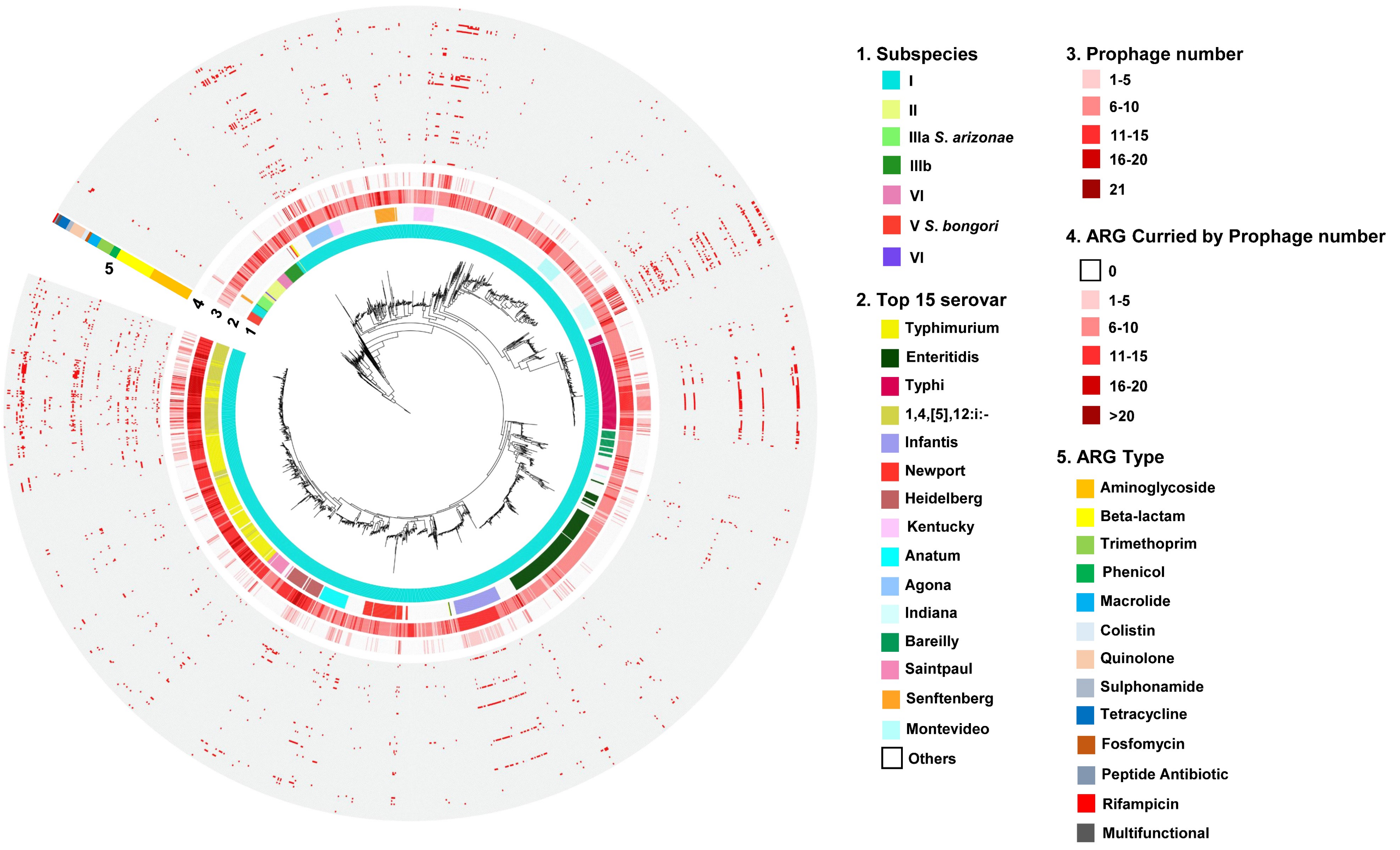

5
4
3
2
1
1. Subspecies
I
II
IIIa S. arizonae
IIIb
VI
V S. bongori
VI
2. Top 15 serovar
Typhimurium
Enteritidis
Typhi
1,4,[5],12:i:-
Infantis
Newport
Heidelberg
Kentucky
Anatum
Agona
Indiana
Bareilly
Saintpaul
Senftenberg
Montevideo
Others
3. Prophage number
1-5
6-10
11-15
16-20
21
4. ARG Curried by Prophage number
0
1-5
6-10
11-15
16-20
>20
5. ARG Type
Aminoglycoside
Beta-lactam
Trimethoprim
Phenicol
Macrolide
Colistin
Quinolone
Sulphonamide
Tetracycline
Fosfomycin
Peptide Antibiotic
Rifampicin
Multifunctional

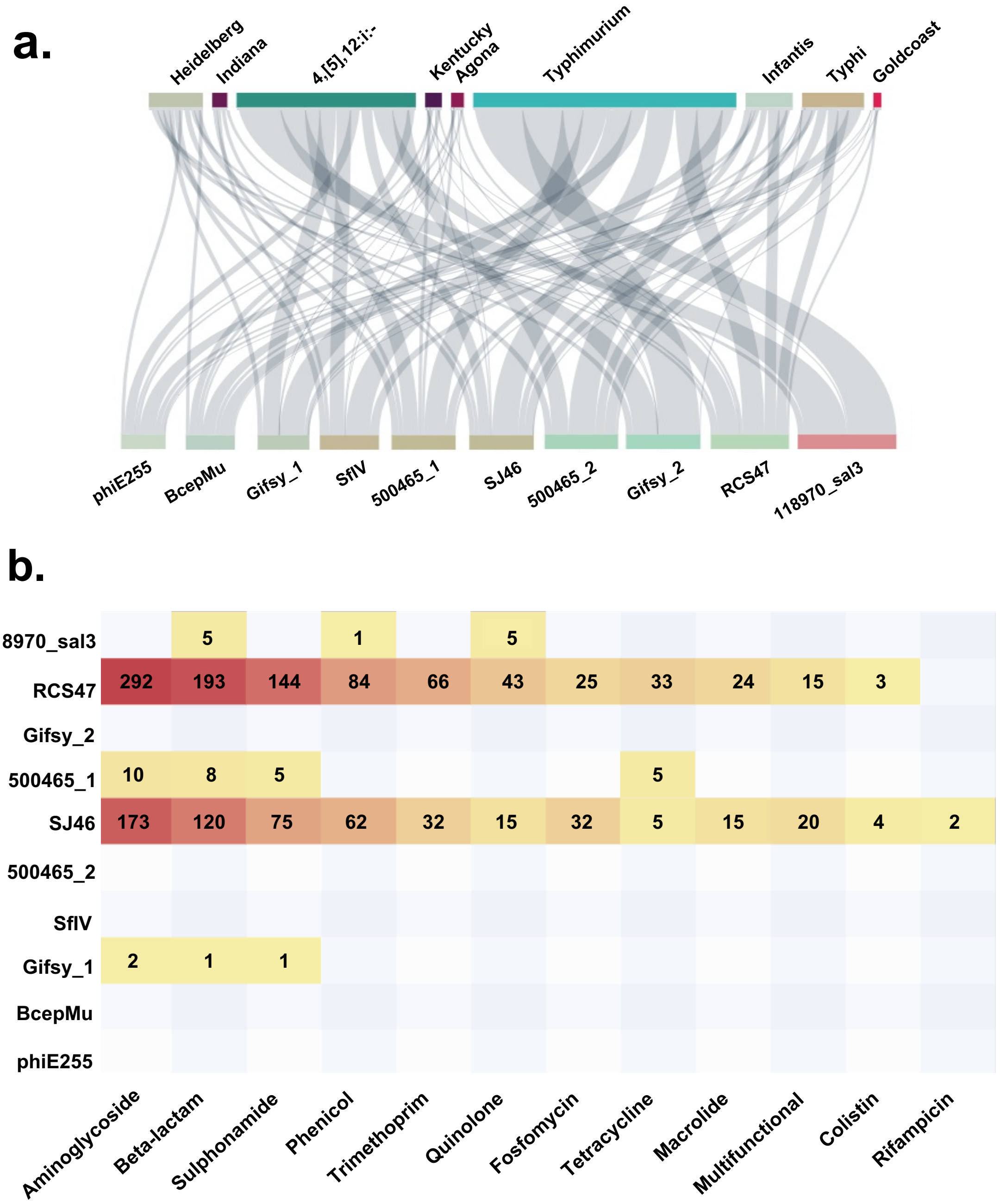
a.
Heidelberg
Indiana
4,[5],12:i:-
Kentucky
Agona
Typhimurium
Infantis
Typhi
Goldcoast
phiE255
BcepMu
Gifsy_1
SfIV
500465_1
SJ46
500465_2
Gifsy_2
RCS47
118970_sal3
b.
118970_sal3 5 1 5
RCS47 292 193 144 84 66 43 25 33 24 15 3
Gifsy_2
500465_1 10 8 5 5
SJ46 173 120 75 62 32 15 32 5 15 20 4 2
500465_2
SfIV
Gifsy_1 2 1 1
BcepMu
phiE255
Aminoglycoside
Beta-lactam
Sulphonamide
Phenicol
Trimethoprim
Quinolone
Fosfomycin
Tetracycline
Macrolide
Multifunctional
Colistin
Rifampicin

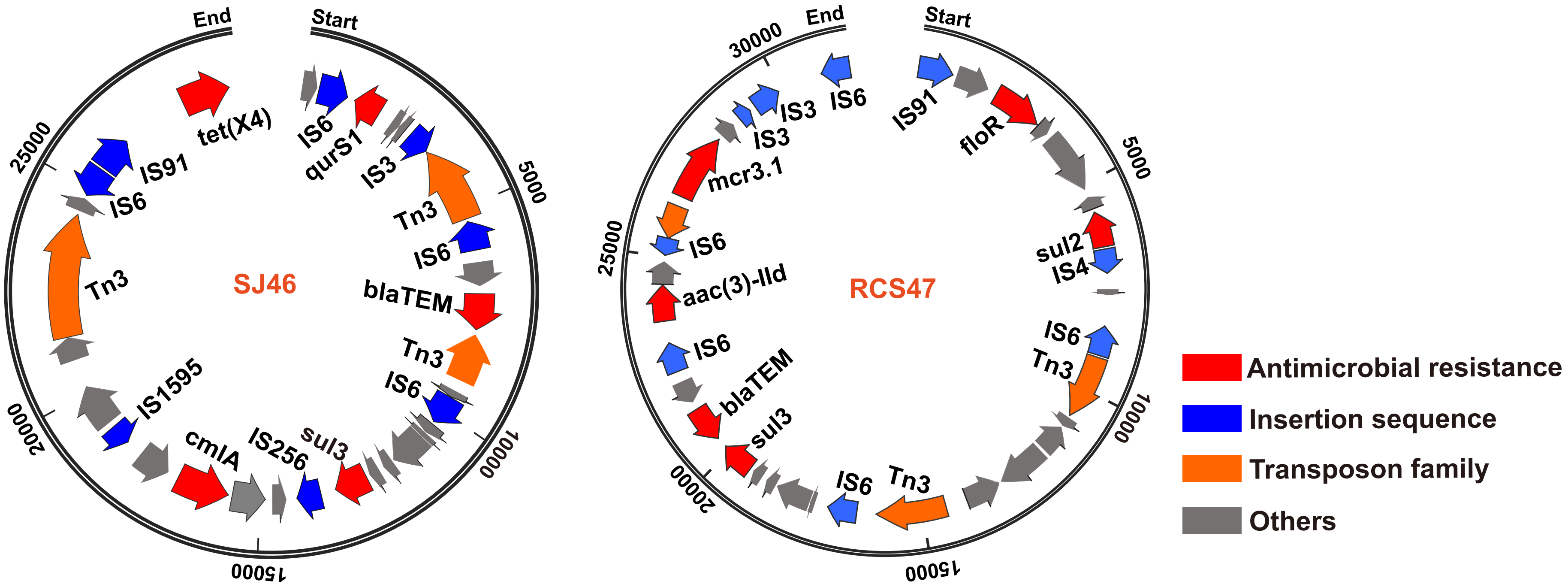
SJ46
Start
End
5000
10000
15000
20000
25000
IS6
qurS1
IS3
Tn3
IS6
blaTEM
Tn3
IS6
sul3
IS256
cmlA
IS1595
Tn3
IS6
IS91
tet(X4)
RCS47
Start
End
5000
10000
15000
20000
25000
30000
IS91
floR
sul2
IS4
IS6
Tn3
Tn3
IS6
sul3
blaTEM
IS6
aac(3)-IId
IS6
mcr3.1
IS3
IS3
IS6
Antimicrobial resistance
Insertion sequence
Transposon family
Others

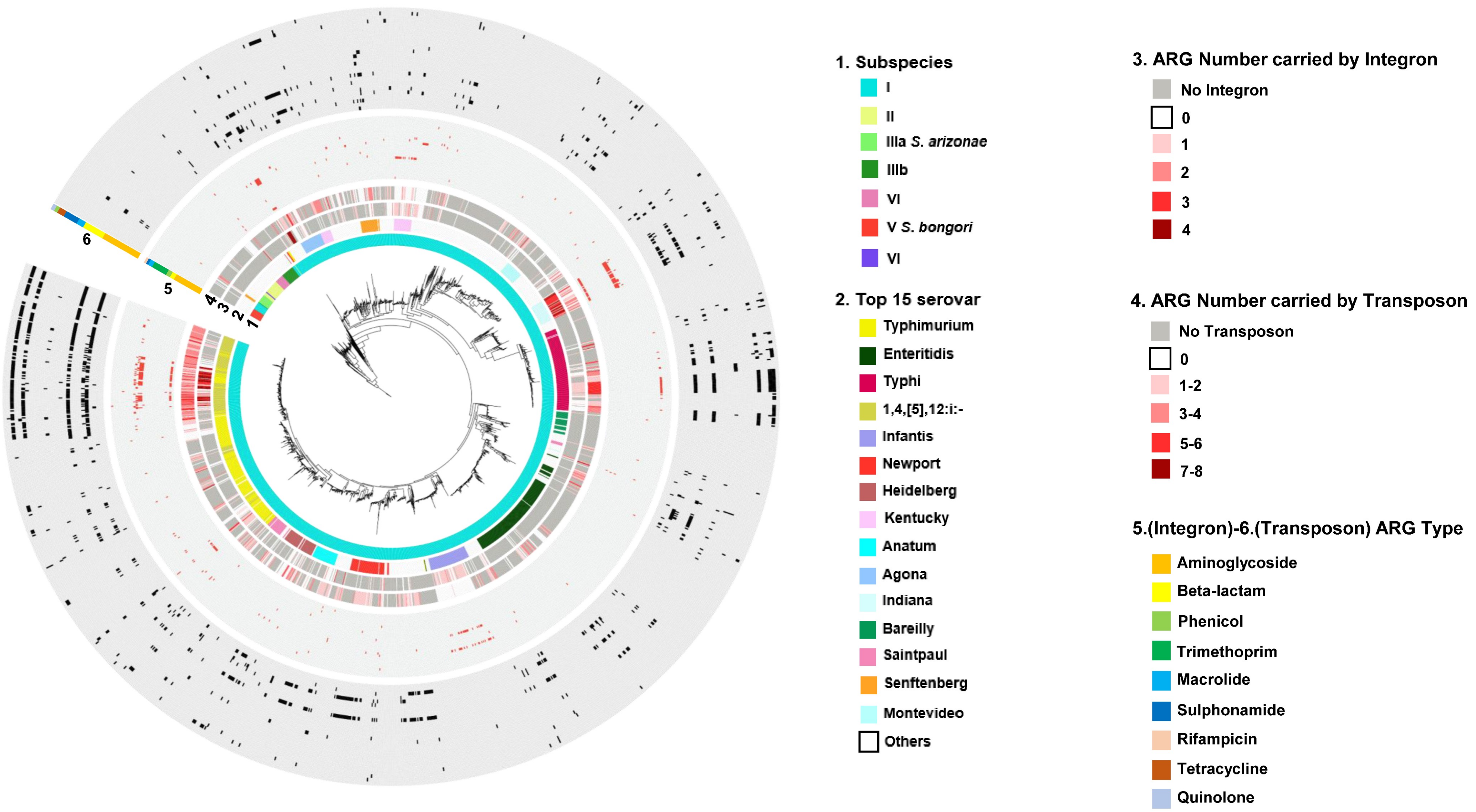

6
5
4
3
2
1
1. Subspecies
I
II
IIIa S. arizonae
IIIb
VI
V S. bongori
VI
2. Top 15 serovar
Typhimurium
Enteritidis
Typhi
1,4,[5],12:i:-
Infantis
Newport
Heidelberg
Kentucky
Anatum
Agona
Indiana
Bareilly
Saintpaul
Senftenberg
Montevideo
Others
3. ARG Number carried by Integron
No Integron
0
1
2
3
4
4. ARG Number carried by Transposon
No Transposon
0
1-2
3-4
5-6
7-8
5.(Integron)-6.(Transposon) ARG Type
Aminoglycoside
Beta-lactam
Phenicol
Trimethoprim
Macrolide
Sulphonamide
Rifampicin
Tetracycline
Quinolone

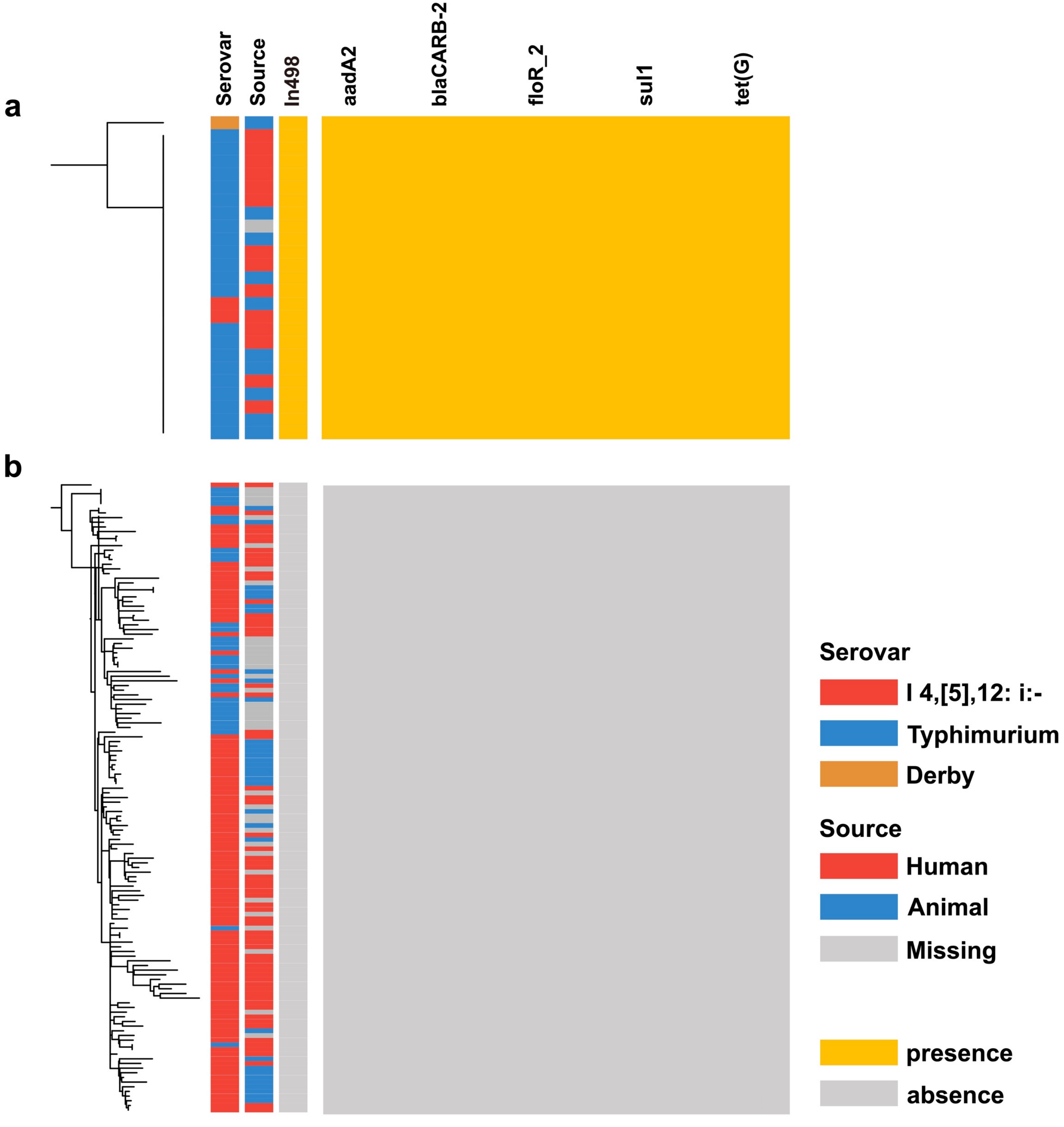

a
b
Serovar
Source
In498
aadA2
blaCARB-2
floR_2
sul1
tet(G)
Serovar
I 4,[5],12: i:-
Typhimurium
Derby
Source
Human
Animal
Missing
presence
absence

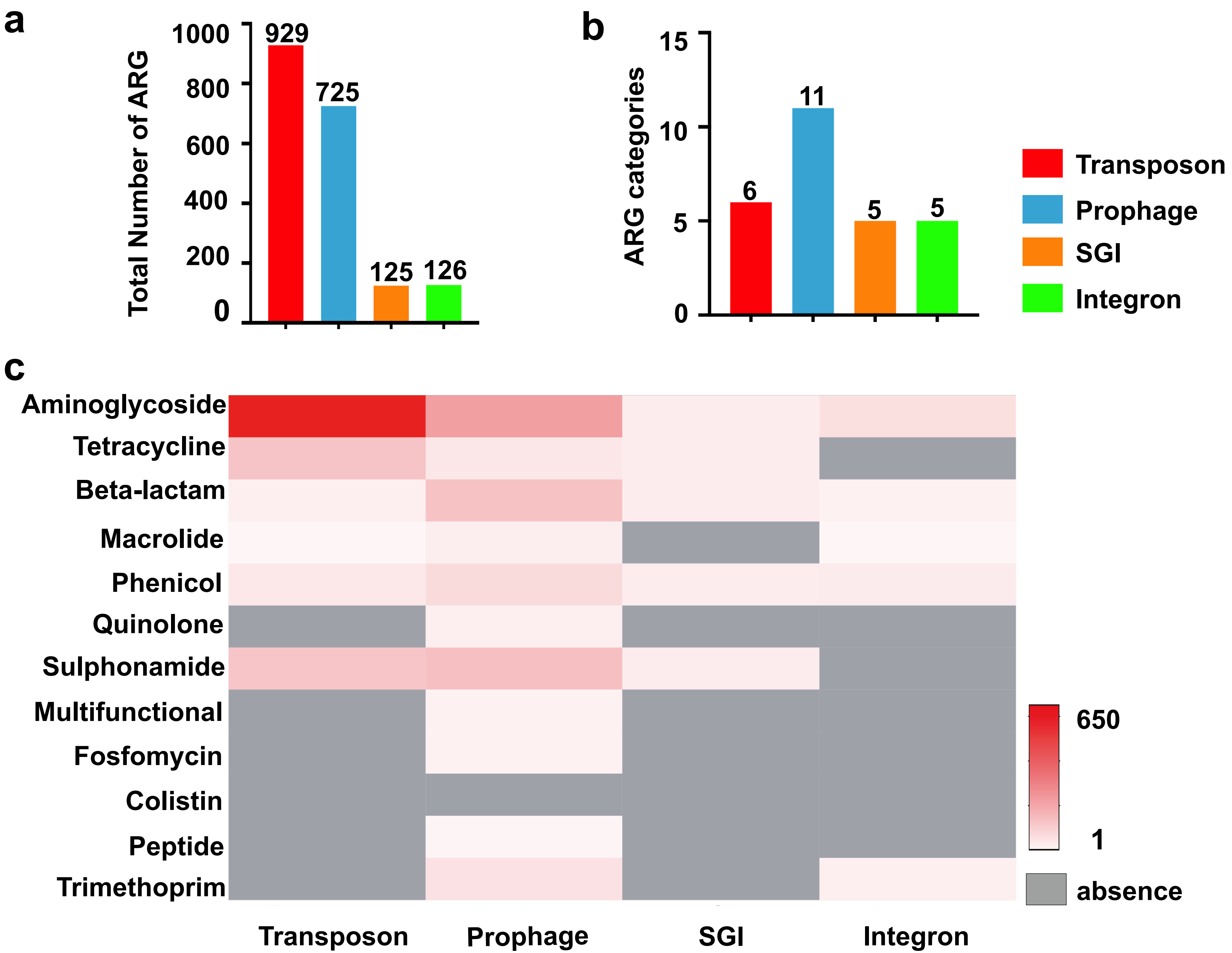
a
b
c
Total Number of ARG
1000
800
600
400
200
0
929
725
125
126
ARG categories
15
10
5
0
6
11
5
5
Transposon
Prophage
SGI
Integron
Aminoglycoside
Tetracycline
Beta-lactam
Macrolide
Phenicol
Quinolone
Sulphonamide
Multifunctional
Fosfomycin
Colistin
Peptide
Trimethoprim
650
1
absence
Transposon
Prophage
SGI
Integron

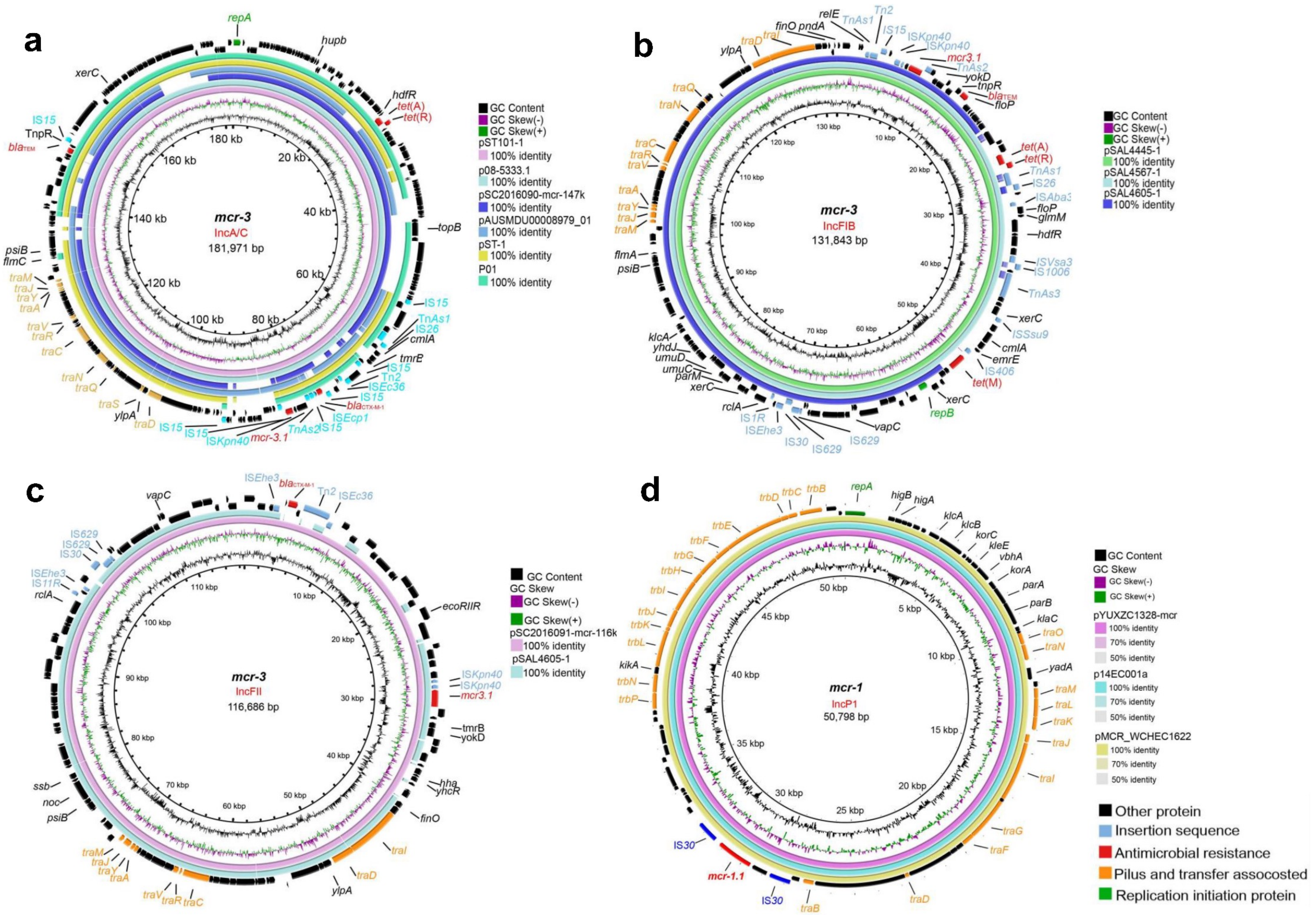
a
repA
hupb
xerC
hdfR
tet(A)
tet(R)
IS15
TnpR
blaTEM
topB
psiB
flmC
traM
traJ
traY
traA
traV
traR
traC
traN
traQ
traS
ylpA
traD
IS15
IS15
ISKpn40
mcr-3.1
TnAs2
IS15
ISEcp1
blaCTX-M-1
IS15
ISEc36
Tn2
IS15
tmrB
cmlA
IS26
TnAs1
IS15
180 kb
160 kb
20 kb
140 kb
40 kb
120 kb
60 kb
100 kb
80 kb
mcr-3
IncA/C
181,971 bp
GC Content
GC Skew(-)
GC Skew(+)
pST101-1
100% identity
p08-5333.1
100% identity
pSC2016090-mcr-147k
100% identity
pAUSMDU00008979_01
100% identity
pST-1
100% identity
P01
100% identity
b
finO
pndA
relE
TnAs1
Tn2
IS15
ISKpn40
ISKpn40
mcr3.1
TnAs2
yokD
tnpR
blaTEM
floP
traD
traI
ylpA
traQ
traN
traC
traR
traV
traA
traY
traJ
traM
flmA
psiB
tet(A)
tet(R)
TnAs1
IS26
ISAba3
floP
glmM
hdfR
ISVsa3
IS1006
TnAs3
xerC
ISSsu9
cmlA
emrE
IS406
tet(M)
xerC
repB
vapC
IS629
IS629
IS30
ISEhe3
IS1R
rclA
xerC
parM
umuC
umuD
yhdJ
klcA
130 kbp
10 kbp
120 kbp
20 kbp
110 kbp
30 kbp
100 kbp
40 kbp
90 kbp
50 kbp
80 kbp
70 kbp
60 kbp
mcr-3
IncFIB
131,843 bp
GC Content
GC Skew(-)
GC Skew(+)
pSAL4445-1
100% identity
pSAL4567-1
100% identity
pSAL4605-1
100% identity
c
vapC
ISEhe3
blaCTX-M-1
Tn2
ISEc36
IS629
IS629
IS30
ISEhe3
IS11R
rclA
ecoRIIR
ISKpn40
ISKpn40
mcr3.1
tmrB
yokD
hha
yhcR
finO
traI
traD
ylpA
traC
traR
traV
traA
traY
traJ
traM
psiB
noc
ssb
110 kbp
10 kbp
100 kbp
20 kbp
90 kbp
30 kbp
80 kbp
40 kbp
70 kbp
50 kbp
60 kbp
mcr-3
IncFII
116,686 bp
GC Content
GC Skew
GC Skew(-)
GC Skew(+)
pSC2016091-mcr-116k
100% identity
pSAL4605-1
100% identity
d
trbD
trbC
trbB
repA
higB
higA
klcA
klcB
korC
kleE
vbhA
korA
parA
parB
klaC
traO
traN
yadA
traM
traL
traK
traJ
traI
traG
traF
traD
traB
IS30
mcr-1.1
IS30
trbE
trbF
trbG
trbH
trbI
trbJ
trbK
trbL
kikA
trbN
trbP
50 kbp
5 kbp
45 kbp
10 kbp
40 kbp
15 kbp
35 kbp
20 kbp
30 kbp
25 kbp
mcr-1
IncP1
50,798 bp
GC Content
GC Skew
GC Skew(-)
GC Skew(+)
pYUXZC1328-mcr
100% identity
70% identity
50% identity
p14EC001a
100% identity
70% identity
50% identity
pMCR_WCHEC1622
100% identity
70% identity
50% identity
Other protein
Insertion sequence
Antimicrobial resistance
Pilus and transfer assocosted
Replication initiation protein

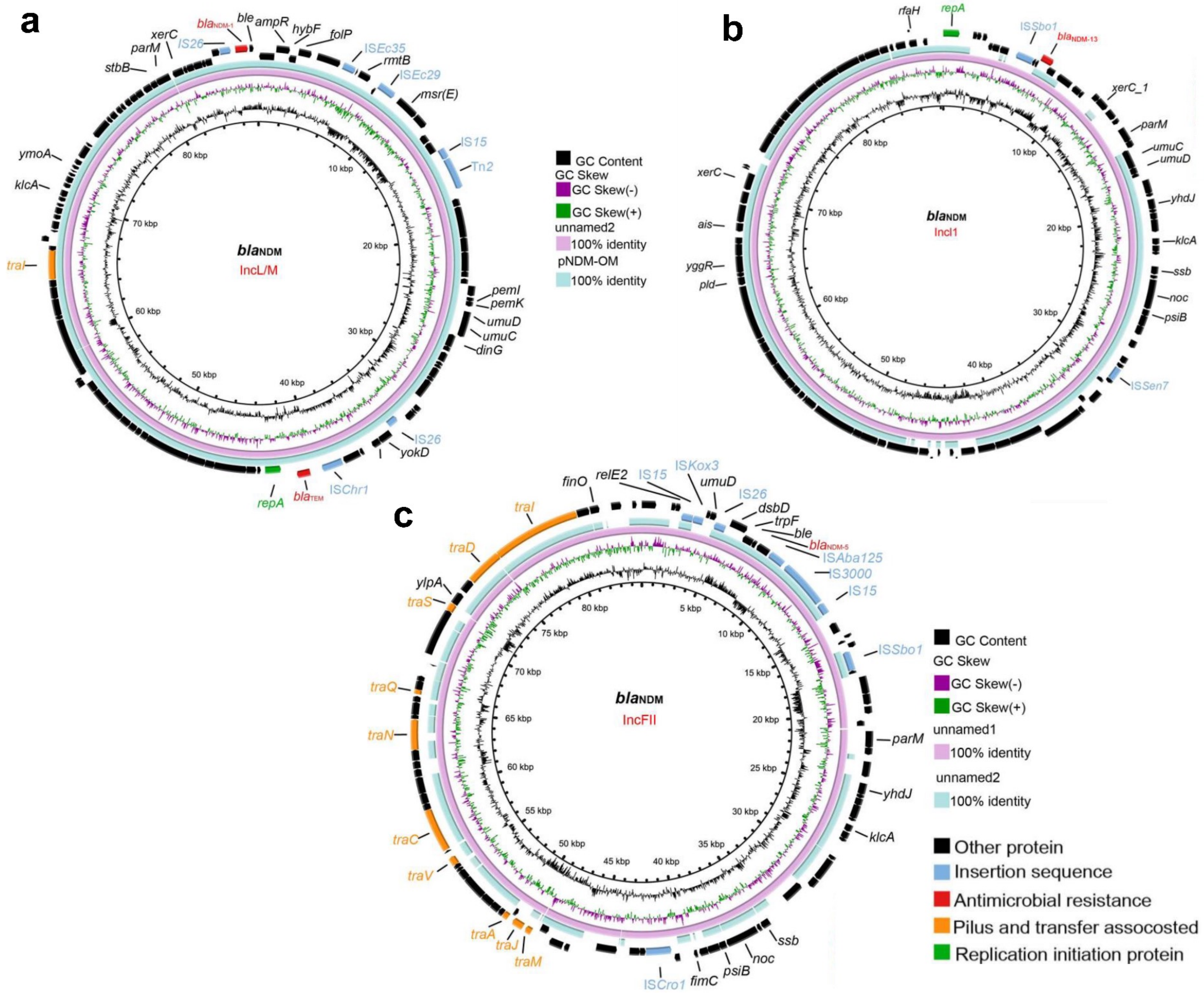
a
blaNDM
IncL/M
blaNDM-1
ble
ampR
hybF
folP
ISEc35
rmtB
ISEc29
msr(E)
IS15
Tn2
pemI
pemK
umuD
umuC
dinG
IS26
yokD
ISChr1
blaTEM
repA
traI
klcA
ymoA
stbB
parM
xerC
IS26
10 kbp
20 kbp
30 kbp
40 kbp
50 kbp
60 kbp
70 kbp
80 kbp
GC Content
GC Skew
GC Skew(-)
GC Skew(+)
unnamed2
100% identity
pNDM-OM
100% identity
b
blaNDM
IncI1
rfaH
repA
ISSbo1
blaNDM-13
xerC_1
parM
umuC
umuD
yhdJ
klcA
ssb
noc
psiB
ISSen7
pld
yggR
ais
xerC
10 kbp
20 kbp
30 kbp
40 kbp
50 kbp
60 kbp
70 kbp
80 kbp
GC Content
GC Skew
GC Skew(-)
GC Skew(+)
pNDM13-SR33
100% identity
pST3606-1
100% identity
c
blaNDM
IncFII
finO
relE2
IS15
ISKox3
umuD
IS26
dsbD
trpF
ble
blaNDM-5
ISAba125
IS3000
IS15
ISSbo1
parM
yhdJ
klcA
ssb
noc
psiB
fimC
ISCro1
traM
traJ
traA
traV
traC
traN
traQ
traS
ylpA
traD
traI
5 kbp
10 kbp
15 kbp
20 kbp
25 kbp
30 kbp
35 kbp
40 kbp
45 kbp
50 kbp
55 kbp
60 kbp
65 kbp
70 kbp
75 kbp
80 kbp
GC Content
GC Skew
GC Skew(-)
GC Skew(+)
unnamed1
100% identity
unnamed2
100% identity
Other protein
Insertion sequence
Antimicrobial resistance
Pilus and transfer assocosted
Replication initiation protein

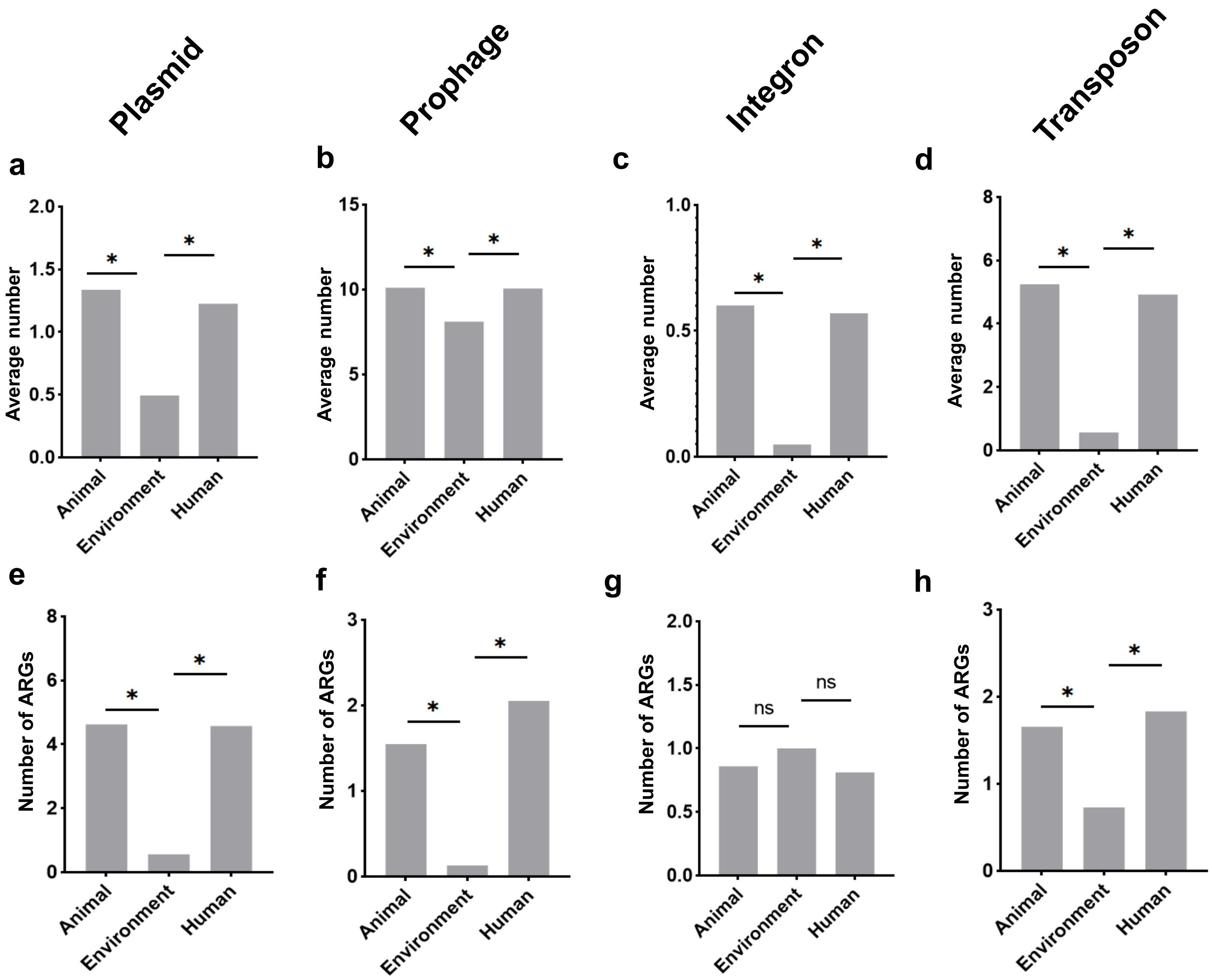

Plasmid
Prophage
Integron
Transposon
a
b
c
d
e
f
g
h
Average number
Number of ARGs
Animal
Environment
Human
*
ns

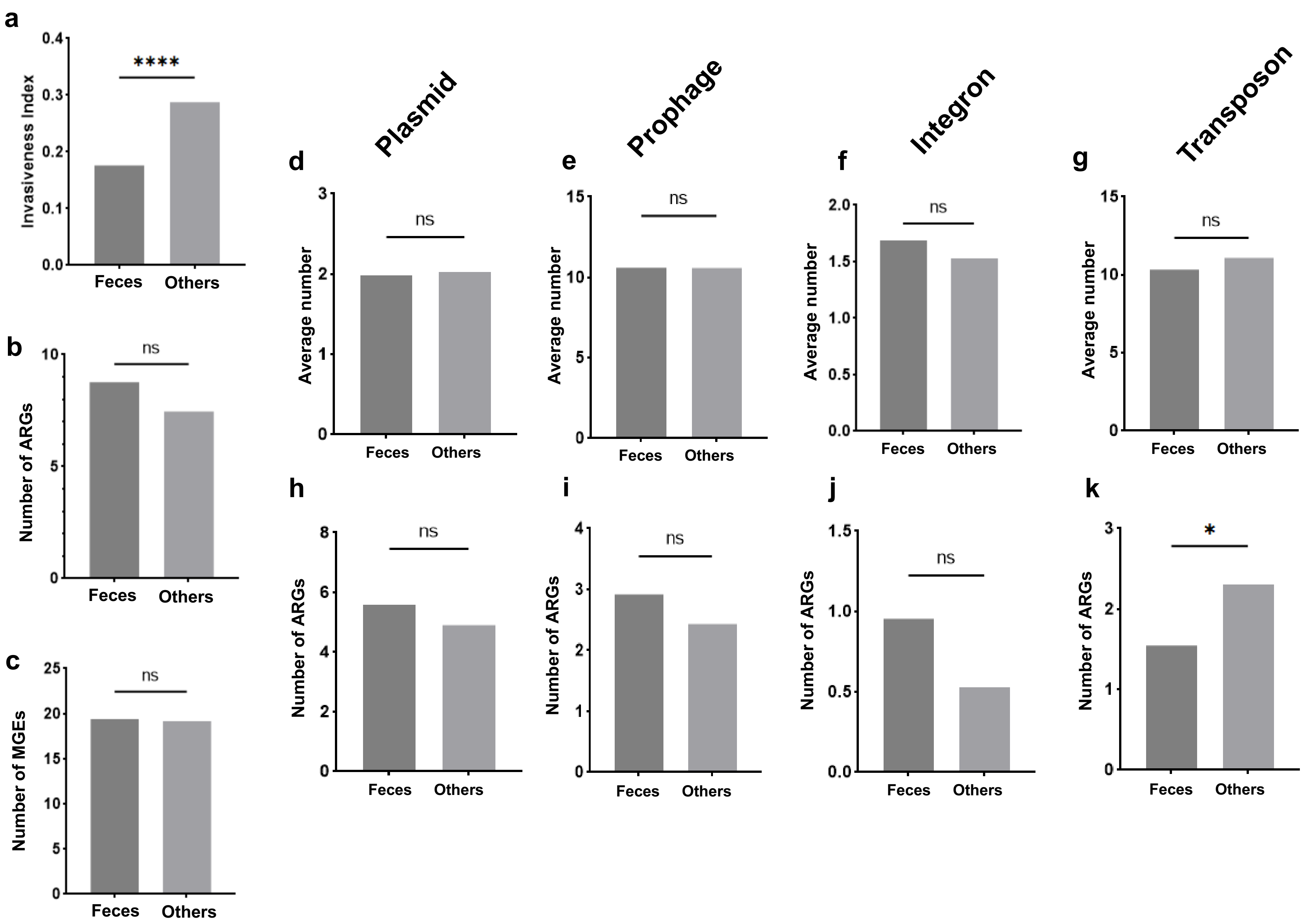

a
Invasiveness Index
0.0
0.1
0.2
0.3
0.4
****
Feces
Others
b
Number of ARGs
0
5
10
ns
Feces
Others
c
Number of MGEs
0
5
10
15
20
25
ns
Feces
Others
Plasmid
Prophage
Integron
Transposon
d
Average number
0
1
2
3
ns
Feces
Others
e
Average number
0
5
10
15
ns
Feces
Others
f
Average number
0.0
0.5
1.0
1.5
2.0
ns
Feces
Others
g
Average number
0
5
10
15
ns
Feces
Others
h
Number of ARGs
0
2
4
6
8
ns
Feces
Others
i
Number of ARGs
0
1
2
3
4
ns
Feces
Others
j
Number of ARGs
0.0
0.5
1.0
1.5
ns
Feces
Others
k
Number of ARGs
0
1
2
3
*
Feces
Others
