# Supplementary material for: Mobilome-driven partitions of the resistome in Salmonella
Source: mSystems. 2023 Oct 19;8(6):e00883-23. doi: 10.1128/msystems.00883-23 (PMC10734508; doi:10.1128/msystems.00883-23)
Supplement: Supplemental Legends — Legends for supplemental figures and tables. [file msystems.00883-23-s0002.pdf]

Supplementary Figures

Figure S1. Characteristics of the *Salmonella* strains included in the dataset (a) The number of complete genomic data for *Salmonella* used in this study. (b) The number of different subspecies strains among the analyzed *Salmonella.* (c) The top 15 serotype categories were identified in the dataset. (d) The presence of four types of MGEs in the strains.

Figure S2. Genomic characteristics of 1, 999 plasmids. (a) The number of plasmids identified as plasmid replicon types (n=1,208) and not defined for a plasmid replicon type (n=791) based on the available database. (b) The top 15 plasmid types. (c) A phylogenetic tree of "*IncFII/IncFIB*" type plasmids has been constructed using core SNPs. The outer circle of the tree is color-coded to represent the respective *Salmonella* serovars.

Figure S3. Genomic characteristics of identified prophages. The identification of the prophage source was based on the isolation information provided by the National Center for Biotechnology Information (NCBI) when the prophage was first reported. (a) The average number of prophages. (b) Percentage of 1,817 *Salmonella* carrying different classes of prophage: *Salmonella* carrying prophage (100%), *Salmonella* carrying intact prophages (96.9%), *Salmonella* carrying more than two intact prophages (59.3%), *Salmonella* carrying defective prophage (100%), *Salmonella* carrying more than two defective prophages (96.4%), and percentage of prophage carried by plasmids (51%). (c) The percentage of prophages derived from the *Enterobacteriaceae* family. (d) The percentage of prophages derived from specific genera within the *Enterobacteriaceae* family. *, $P < 0.05$.

Figure S4. Comparison of ARG carriage on plasmids and other MGEs. The heatmap illustrates the quantity of ARGs in each respective category, with darker shades of red indicating a higher number of ARGs carried.

Figure S5. ARGs carried on four types of MGEs. (a) ARGs carried on plasmids (b) ARGs carried by prophages (c) ARGs carried by integrons (d) ARGs carried by transposons.

Figure S6. Plasmid- borne ARGs prevalence among eight key serovars. (a) The main plasmid types carried by key serovar *Salmonella*. (b) The ARGs located on different types of plasmids. * Plasmids carrying T4SS.

Figure S7. Phylogenetic tree of 1,817 *Salmonella*. The first ring of the tree represents the subspecies of each strain, while the second ring shows the different serovars of *Salmonella.* The third ring displays the number of *Salmonella*-carrying prophages, and the fourth ring indicates the number of prophages-bearing ARGs. The fifth ring classifies the ARGs carried on prophages and is color-coded to indicate the presence (in red) or absence (in light grey) of ARGs.

Figure S8. Prophage- borne ARGs prevalence among nine key serovars. (a) The main prophage types prevalent in nine serovars. (b) The ARGs located on different types of prophages.

Figure S9. Comparison of ARG position carried on SJ46 and RCS47.

Figure S10. Phylogenetic tree of 1,817 *Salmonella*. The subspecies of each strain (Ring 1), Different serovars of each strain (Ring 2). The number of

integrons carrying ARGs (Ring 3) and transposons carrying ARGs (Ring 4). The classification of the ARGs is carried on integrons and is color-coded to indicate the presence (in red) or absence (in light grey) of ARGs (RING 5). The classifies the ARGs carried on transposons and is color-coded to indicate the presence (in black) or absence (in light grey) of ARGs (RING 6).

Figure S11. Presence of SGI and SGI-borne ARG in different *Salmonella* serovars. (a). A phylogenetic tree was constructed based on the single nucleotide polymorphisms (SNPs) of *Salmonella* carrying SGI-1 and SGI-1 variants. The Heatmap on the right presents the *Salmonella* serovar, isolate source, integron and ARG types located on the SGI, respectively. (b). A phylogenetic tree was constructed based on the SNPs of *Salmonella* carrying SGI-4. The Heatmap on the right presents the *Salmonella* serovar, isolate source, integron and ARG types located on the SGI-4, respectively.

Figure S12. The ARGs are carried by four types of MGEs (transposons, prophages, SGI, and integrons) located on the chromosome. (a). Total number of ARGs carried by each type of MGE. (b). Number of ARG categories carried by each type of MGE. (c). Heatmap of the categories and number of ARGs carried by each type of MGE.

Figure S13. Plasmid characteristics and comparison of the plasmids harboring *mcr* family ARGs using BRIG.

Figure S14. Plasmid characteristics and comparison of the plasmids harboring *bla*$_{\text{NDM}}$ family ARGs using BRIG.

Figure S15. Association analysis of *Salmonella* carrying MGEs and the average number of ARGs on MGEs with three different isolate sources (Animal/Environment/Human). (a-d) The average number of identified MGEs carried by each *Salmonella* was categorized by the source of the isolate. (e-h) The average number of ARGs carried on the identified MGEs, categorized by the source of the isolate. *, $P < 0.05$.

Figure S16. Association analysis of *Salmonella* carrying MGEs and the average number of ARGs on MGEs with two different isolate sources (Human feces/Human, non-fecal source). (a) The invasiveness index value for *Salmonella* from two different sample sources. (b) The average number of *Salmonella* from the two different sources that carry all four MGEs. (c) The average number of ARGs carried overall on the four MGEs. (d-g) The average number of MGEs carried by *Salmonella* is categorized by the two different sources. (h-k) The average number of ARGs carried by *Salmonella* is categorized by the two different sources. *, $P < 0.05$. ****, $P < 0.001$.

Supplementary Tables

Table S1. Summary of 1,817 complete *Salmonella* genome sequences collected from GenBank and the GDP per capita of the isolating country in 2022.

Table S2. Details of the four MGEs (plasmid, prophage, integron, transposon) detected on 1,817 *Salmonella*.

Table S3. Information on plasmids and prophages carrying $\mathit{bla}_{\mathrm{NDM}}$, *mcr*, and *tet*(X) family ARGs, and serovars of *Salmonella* carrying $\mathit{bla}_{\mathrm{NDM}}$, *mcr*, and *tet*(X) family ARGs.

Table S4. Chromosomes and plasmids- borne ARGs of 1,817 *Salmonella*, and the ARGs located on four types of chromosomes- carrying MGE.
